# Genomic epidemiological analysis identifies high relapse among individuals with recurring tuberculosis and provides evidence of recent household-related transmission of tuberculosis in Ghana

**DOI:** 10.1016/j.ijid.2021.02.110

**Published:** 2021-05

**Authors:** Prince Asare, Stephen Osei-Wusu, Nyonuku Akosua Baddoo, Edmund Bedeley, Isaac Darko Otchere, Daniela Brites, Chloé Loiseau, Adwoa Asante-Poku, Diana Ahu Prah, Sonia Borrell, Miriam Reinhard, Michael Amo Omari, Audrey Forson, Kwadwo Ansah Koram, Sebastien Gagneux, Dorothy Yeboah-Manu

**Affiliations:** a Noguchi Memorial Institute for Medical Research, College of Health Sciences, University of Ghana (UG), Ghana; b West African Centre for Cell Biology of Infectious Pathogens, UG, Ghana; c Department of Biochemistry, Cell and Molecular Biology, UG, Ghana; d Department of Chest Diseases, Korle-Bu Teaching Hospital, Korle-Bu, Accra, Ghana; e Swiss Tropical and Public Health Institute, Basel, Switzerland; f University of Basel, Basel, Switzerland

**Keywords:** Tuberculosis, *Mycobacterium tuberculosis*, *Mycobacterium africanum*, Molecular epidemiology, Whole-genome sequencing, Relapse

## Abstract

**Objective:**

To retrospectively investigate the cause of recurring tuberculosis (*rc*TB) among participants with pulmonary TB recruited from a prospective population-based study conducted between July 2012 and December 2015.

**Methods:**

*Mycobacterium tuberculosis* complex isolates obtained from *rc*TB cases were characterized by standard mycobacterial genotyping tools, whole-genome sequencing, and phylogenetic analysis carried out to assess strain relatedness.

**Results:**

The majority (58.3%, 21/36) of study participants with *rc*TB episodes had TB recurrence within 12 months post treatment. TB strains with isoniazid (INH) resistance were found in 19.4% (7/36) of participants at the primary episode, of which 29% (2/7) were also rifampicin-resistant. On TB recurrence, an INH-resistant strain was found in a larger proportion of participants, 27.8% (10/36), of which 40% (4/10) were MDR-TB strains. *rc*TB was attributed to relapse (same strain) in 75.0% (27/36) of participants and 25.0% (9/36) to re-infection.

**Conclusion:**

Our findings indicate that previous unresolved infectiondue to inadequate treatment, may be the major cause of *rc*TB.

## Highlights

• Unresolved previous infection as major cause of recurring tuberculosis (TB) in Ghana.
• Genomic epidemiology identifies high relapse among recurrent TB cases in Ghana.
• 15-locus MIRU-VNTR typing is sufficient to predict the cause of TB recurrence.
• Evidence of recent household-related TB transmission in Ghana.
• Need for increased education by national TB control program.

## Introduction

Tuberculosis (TB) remains a major global public health threat (WHO, 2018a); thus, more efforts are needed to deal with this global problem. Despite the effective use of combination therapy in the directly observed treatment short-course (DOTS) regimen since 1993 (Alipanah et al., 2018), some previously treated patients still present with a secondary case of the disease, here referred to as recurring TB (*rc*TB). Recurring TB is characterized by the return of symptoms in a patient declared cured or having completed treatment. It is important to distinguish recurrence due to relapse caused by the initial strain (endogenous reactivation of previous infection) from reinfection with a new strain (exogenous reinfection). The former indicates unsuccessful therapy, while the latter indicates patients’ elevated susceptibility to the disease and/or chronic exposure to the bacilli and usually occurs among HIV co-infected patients and those living in countries with high TB burden (Narayanan et al., 2010). Whereas reinfection has been identified as the principal cause of *rc*TB in high TB burden areas (Parvaresh et al., 2018, van Rie et al., 1999), relapse may be the result of poor prognosis and unsuccessful treatment (Alipanah et al., 2018) and has been associated with drug resistance (Yang et al., 2017).

Traditional typing methods such as mycobacterial interspersed repetitive-unit variable-number tandem-repeat (MIRU-VNTR) and spoligotyping have been used extensively to determine strain relatedness (Velayutham et al., 2018, Zong et al., 2018). However, whole-genome sequencing (WGS), considered the ultimate tool for strain differentiation, has not been widely explored in low resource settings due to the high cost and technological demands. WGS data allows us to identify recent TB transmission between individuals of the same households and trace the route/direction of transmission between such epidemiologically linked cases (Walker et al., 2013). This study aimed to identify and delineate the occurrence of *rc*TB episodes and confirm transmission among epidemiologically linked cases in distinct Ghanaian communities using WGS.

## Methods

### Study design and population

**Figure 1:**
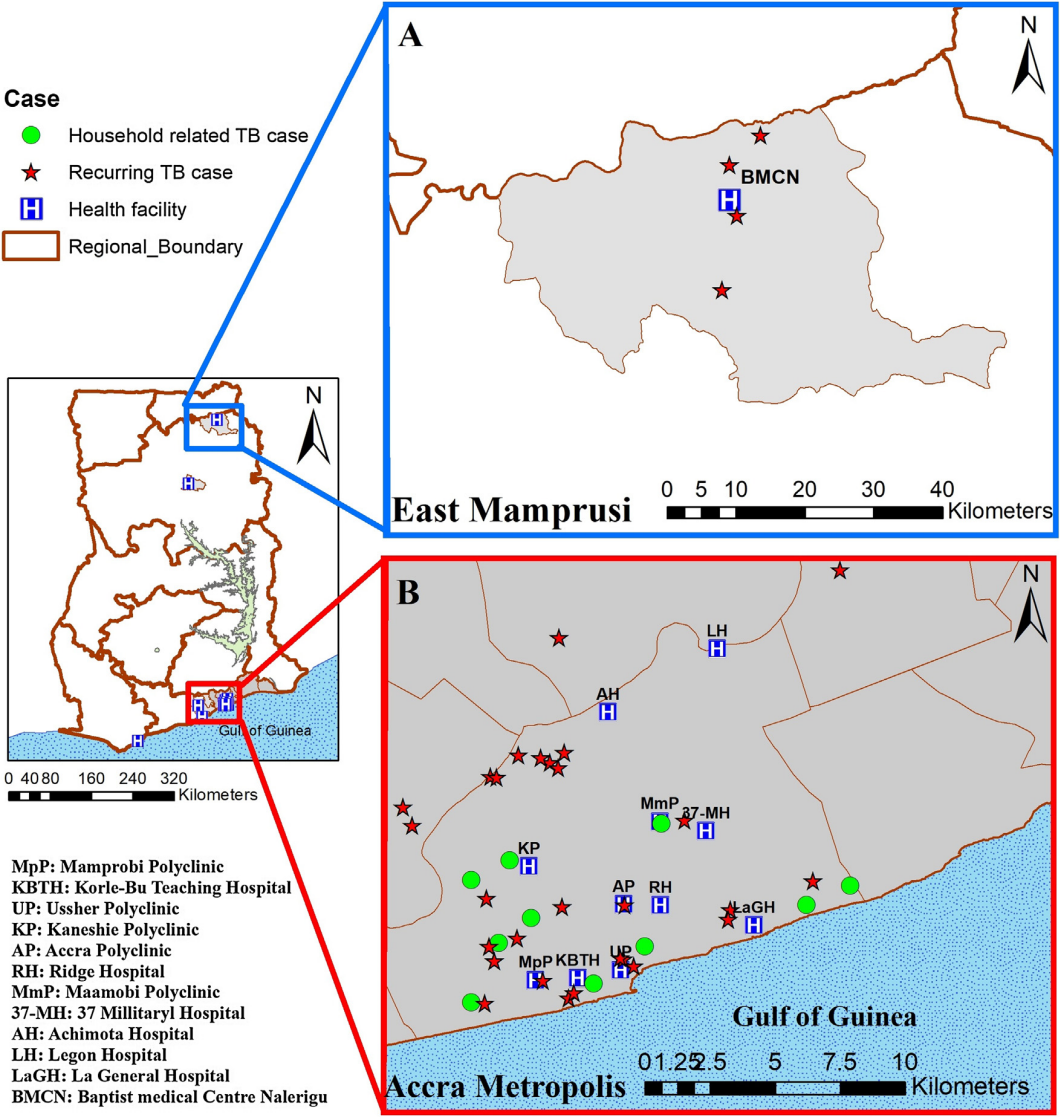
Relative geographical location of 13 health facilities and distribution of recurring and household-related TB cases. Participant recruitment was carried out in 2 areas, the East Mamprusi District (A) and Accra Metropolitan Area (B).

The study was a retrospective analysis of *Mycobacterium tuberculosis* complex (MTBC) isolates from a population-based study that recruited >90% of TB cases from the Accra Metropolitan Area (urban/south) and the Mamprusi East (rural/north) for >3 years (Figure 1) (Asare et al., 2018, Yeboah-Manu et al., 2016). All individuals who presented with >1 TB episode (>6 months between visits) post initial treatment for TB were included; herein referred to as *rc*TB cases (Thomas et al., 2005, Velayutham et al., 2018). We also included all cases belonging to the same household (epidemiologically linked) for households with >1 TB case; herein referred to as household-related TB cases. The cases' clinical and sociodemographic characteristics were obtained from medical records and a detailed questionnaire after obtaining informed written consent from each participant. We obtained permission from the guardians of participants <18 years old. All participants were treated following the DOTS regimen using a combination of 4 first-line antibiotics; isoniazid (INH), rifampicin (RIF), ethambutol (EMB) and pyrazinamide (PZA).

### Mycobacterial isolation, DNA extraction, characterization and drug susceptibility testing

Mycobacterial isolates were obtained from sputum samples cultured on Lowenstein Jensen media and characterized as previously described (Yeboah-Manu et al., 2016). Genomic DNA extraction for genome sequencing was performed using the cetyl trimethyl ammonium bromide (CTAB) protocol (Otchere et al., 2016) with one amendment: to obtain sufficient intact genomic DNA (gDNA), harvested mycobacterial cells were heat-inactivated at 80 °C for 30 min (instead of 95 °C for 1 h) in the cell lysis buffer (Supplementary Figure 1).

### Traditional strain typing and drug susceptibility testing

Traditional strain typing was performed using the standard 15-MIRU loci set (Supply et al., 2006), and drug susceptibility testing carried out as previously described (Asare et al., 2018). In addition to the *in vitro* tests, *in silico* analyses were performed using the TBprofiler package (Coll et al., 2015) to detect mutations associated with drug resistance and report other mutations present in drug-resistant associated genes.

### Whole-genome sequencing and analysis

Illumina sequencing libraries were prepared using NEBNEXT ULTRA II FS DNA library preparation kit (New England Biolabs), then multiplexed paired-end/single-end sequencing was performed using the HiSeq2500 NGS platform (Illumina, San Diego, CA, United States). Information on raw sequence data (registered under BioProject ID: PRJNA616081) is provided in Supplementary Table 1. The sequence reads were quality controlled and analyzed using previously described procedures (Brites et al., 2018).

### Phylogenetic reconstruction

Customized python scripts were used to produce separate multifasta alignment files for genomes from *rc*TB and household-related cases. Only polymorphic positions were included for phylogenetic reconstruction analysis after excluding genomic positions with >10% missing calls. The GTR-GAMMA model with 1000 rapid bootstrap inferences, followed by a thorough maximum-likelihood search performed in CIPRES (Miller et al., 2010), was used to infer a maximum likelihood phylogenetic tree using RaxML v8.2.3 (Stamatakis, 2014). All phylogenetic trees were reconstructed and annotated using the ggtree package in R (Yu et al., 2018) and graphics enhanced using ggplot2 (Wickham, 2016) also implemented in R (R, 2019) (http://cran.r-project.org/). Pairwise SNP distances were calculated between each pair of genomes from the same participant using Mega v10.0.5 (Kumar et al., 2018).

### Case definitions

A case was defined as relapse when MTBC isolates recovered from both episodes had < = 1 allelic difference in their MIRU profile and < = 10 SNP differences between their respective genomes (Walker et al., 2013). Conversely, we defined reinfection when there was >1 allelic repeat difference in MIRU profiles and >50 SNPs between genomes (Walker et al., 2013).

### Data analysis and epidemiology

Data obtained using the structured questionnaire were double examined for completeness and consistency and entered in Microsoft Access. All statistical analysis was performed using the Stata statistical package version 14.2 (Stata Corp., College Station, TX, USA) and run with a significance level of *P* < 0.05 using Fischer’s exact test. We used the Kappa test to test for concordance between typing methods. The ArcMap tool employed in ArcGIS (Economic and Social Research Institute, version 10.1) (ESRI, 2010) was used for constructing maps.

This study is reported according to the Strengthening the Reporting of Molecular Epidemiology for Infectious Diseases (STROME-ID) guidelines (Field et al., 2014).

## Results

**Figure 2:**
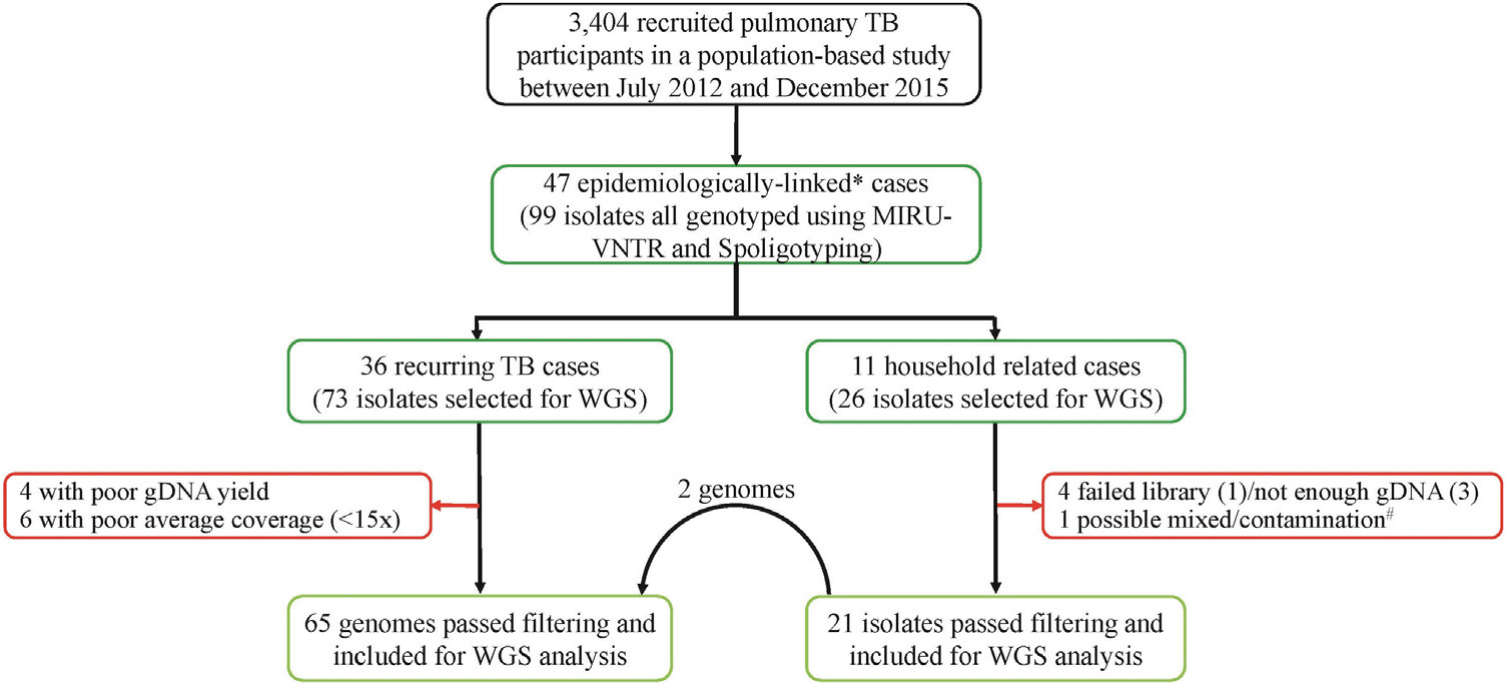
Pipeline for sample selection and genotyping. *Isolates were classified as epidemiologically linked if they came from the same household or the same participant (longitudinal isolates). ^#^Genomes with heterozygous SNPs >120 were classified as possible mixed infection or contamination and hence removed from further analysis. ^†^Excluded due to unavailable genome for isolate pairs. MIRU-VNTR: mycobacteria interspersed repetitive-unit variable-number tandem-repeat typing, WGS: whole genome sequencing, gDNA: genomic DNA.

We included in our analysis 99 MTBC isolates from 47 TB cases, of which 36 cases (75 isolates) had *rc*TB and 11 (26 isolates) were suspected household-related transmissions (Figure 2). One individual (2 isolates) involved in a household-related TB case also had *rc*TB and was included in both analyses.

### Characteristics of individuals with recurring TB episodes

The majority (94.4%, 34/36) of participants with *rc*TB episodes were male. The age range was 22-75 years, with a median age of 39.5 (IQR, 32-53). Of the 36 participants, 21 (58.6%) had TB recurrence within 12 months post treatment. All 36 *rc*TB cases had either been cured (11), completed treatment (4), defaulted (3), or there was no data available (18) on the outcome of the primary episode. Twenty-four (66.7%) participants had a smear grade >1+ at the primary episode. Of the participants tested for co-infections, 8.3% (2/24) and 36.8% (7/22) were positive for HIV and diabetes mellitus, respectively. A strain with INH resistance was found in 19.4% (7/36) of participants at the primary episode, of which 29% (2/7) were also resistant to RIF (multi-drug-resistant TB [MDR-TB]). Among the recurring episodes, INH-resistant strains were found in 27.8% (10/36) of participants, of which 40% (4/10) were MDR-TB strains (Table 1). Two of the 4 MDR-TB cases had recurrence with different strains, while the remaining 2 were the same strains identified from the previous episode. In two cases (RL009 and RL019) with the same strain at both episodes, the strain was INH-sensitive at the first episode and resistant at the second episode. The majority of cases were MTBC lineages belonging to *Mycobacterium tuberculosis* sensu stricto (MTBss) lineages 2 or 4 (77.8%, 28/36), with the Cameroon sublineage causing 50% (14/28) of such cases in both episodes, followed by the Ghana sublineage (17.9%, 5/28).

**Table 1:** Characteristics of cases with recurring TB episodes.

Participant ID	Gender	Age	Smear grade		Duration between follow-up (days)	MTBC lineage/spoligotype		Strain similarity^a^	Outcome of previous treatment	Conventional DR 1°		Conventional DR 2°/3°		Genotypic DR (mutation^b^) 1°		Genotypic DR (mutation^b^) 2°/3°		Pairwise SNP distance	WGS status
										INH	RIF	INH	RIF	INH	RIF	INH	RIF		
			1°	2°/3°		1°	2°/3°												
RL001	Male	26	3+	3+	397	L4/Cameroon	L4/Cameroon	Same	cured	S	S	S	S	S	S	S	S	0	Available
RL002	Male	48	2+	2+	189	L4/Haarlem	L4/Haarlem	Same	NA	S	S	S	S	S	S	S	S	1	Available
RL003	Male	27	3+	1+	707	L6/West African 2	L4/Cameroon	Different	Completed	S	S	S	S	S	S	S	S	1848	Available
RL004	Male	34	1+	scanty	624	L4/Cameroon	L4/Cameroon	Same	NA	S	S	S	S	NA	NA	S	S	NA	Available for 1
RL005	Male	52	1+	3+	196	L5/West African 1	L5/West African 1	Different	cured	R	S	R	R	S	S	R (-15C > T/fabG1/ Rv1483)	S	334	Available
RL006	Male	22	1+	3+	161	L4/Ghana	L6/West African 2	Different	Completed	S	S	S	S	S	S	NA	NA	NA	Available for 1
RL007	Female	25	3+	2+	603	L5/West African 1	L5/West African 1	Different	defaulted	R	S	R	S	S	S	R (-15C > T/fabG1/ Rv1483)	R (L452 P/rpoB/Rv0667)	243	Available
RL008	Male	40	3+	1+	476	L4/Ghana	L4/Cameroon	Different	NA	S	S	S	S	S	S	S	S	571	Available
RL009	Male	NA	1+	3+	279	L4/Cameroon	L4/Cameroon	Same	NA	S	S	R	S	S	S	S	S	0	Available
RL010	Female	24	2+	1+	570	L4/Cameroon	L4/Cameroon	Same	NA	S	S	S	S	S	S	S	S	7	Available
RL011	Male	53	1+	1 /3+	231/532	L4/Cameroon	L4/Cameroon	Same	defaulted	S	S	S	S	S	S	S	S	0/0/0	Available
RL012	Male	70	1+	3+	524	L2/Beijing	L2/Beijing	Same	NA	S	S	S	S	S	S	S	S	0	Available
RL013	Male	55	1+	NA/2+	164/408	L4/Cameroon	L4/Cameroon	Same	Completed	S	S	S	S	S	S	S	S	0	Available for 2
RL014	Male	35	3+	3+	484	L4/Cameroon	L4/Cameroon	Same	defaulted	S	S	S	S	S	S	S	S	0	Available
RL015	Male	58	3+	3+	237	L4/Cameroon	L4/Ghana	Different	NA	S	S	S	S	S	S	S	S	655	Available
RL016	Male	31	1+	3+	700	L6/West African 2	L6/West African 2	Same	cured	R	S	R	S	NA	NA	R (S315 T/KatG/Rv1908c)	R(L452 P/rpoB/Rv0667)	NA	Available for 1
RL017	Male	75	1+	scanty	252	L4/Ghana	L4/Ghana	Same	cured	S	S	S	S	NA	NA	NA	NA	NA	NA
RL018	Male	52	1+	NA	519	L4/Haarlem	L4/Ghana	Different	Completed	R	S	R	R	NA	NA	NA	NA	NA	NA
RL019	Male	68	3+	scanty	595	L4/Ghana	L4/Ghana	Same	cured	S	S	R	S	S	S	NA	NA	NA	Available for 1
RL020	Male	43	3+	3+	263	L4/Haarlem	L4/Haarlem	Same	cured	S	S	S	S	S	S	S	S	0	Available
RL021	Male	52	3+	1+	413	L4/Cameroon	L4/Cameroon	Same	cured	S	S	S	S	NA	NA	S	S	NA	Available for 1
RL022	Male	37	3+	3+/3+	350/476	L4/Cameroon	L4/Cameroon	Same	NA	S	S	S	S	S	S	S	S	0/0/0	Available
RL023	Male	53	2+	2+	419	L4/Ghana	L4/Ghana	Same	cured	R	S	R	S	R (S315 T/KatG/Rv1908c)	S	R (S315 T/KatG/Rv1908c)	S	0	Available
RL024	Male	32	1+	2+	196	L4/Cameroon	L4/Cameroon	Same	NA	S	S	S	S	S	S	S	S	0	Available
RL025	Male	27	2+	scanty	271	L4/Ghana	L4/Ghana	Same	NA	R	R	R	R	R (S315 T/KatG/Rv1908c)	R (D435 V/rpoB/Rv0667)	R (S315 T/KatG/Rv1908c)	R (D435 V/rpoB/Rv0667)	2	Available
RL026	Male	34	2+	2+	329	L4/Cameroon	L4/Cameroon	Different	cured	S	S	R	S	S	R (S450 L,rpoB,Rv0667)	R (-15C > T/fabG1/ Rv1483)	S	62	Available
RL027	Male	39	1+	NA	314	L5/West African 1	L5/West African 1	Same	NA	S	S	S	S	S	S	S	S	0	Available
RL028	Male	59	1+	NA	371	L5/West African 1	L5/West African 1	Same	cured	R	R	R	R	R (S315 T/KatG/Rv1908c)	R (Q432E/rpoB/Rv0667)	R (S315 T/KatG/Rv1908c)	R (Q432E/rpoB/Rv0667)	0	Available
RL029	Male	70	3+	NA	383	L4/Ghana	L4/Ghana	Same	NA	S	S	S	S	S	S	S	S	0	Available
RL030	Male	34	2+	NA	232	L4/Cameroon	L4/Cameroon	Same	NA	S	S	S	S	S	S	S	S	0	Available
RL031	Male	32	3+	NA	352	L5/West African 1	L5/West African 1	Same	NA	S	S	S	S	S	S	S	S	0	Available
RL032	Male	37	3+	NA	288	L4/Cameroon	L4/Cameroon	Same	NA	S	S	S	S	S	S	S	S	0	Available
RL033	Male	68	2+	NA	338	L4/Cameroon	L4/Cameroon	Same	NA	S	S	S	S	S	S	S	S	5	Available
RL034	Male	25	3+	NA	268	L4/Ghana	L5/West African 1	Different	NA	S	S	S	S	S	S	S	S	1749	Available
RL035	Male	36	3+	NA	252	L2/Beijing	L2/Beijing	Same	NA	S	S	S	S	S	S	S	S	0	Available
RL036	Male	50	2+	2+	237	L4/Haarlem	L4Haarlem	Same	cured	S	S	S	S	S	S	S	S	0	Available

We obtained 3 longitudinal isolates from each of recurrent cases RL011, RL013 and RL022

NA: not available, WGS: whole-genome sequence, SNP: single nucleotide polymorphism, INH: isoniazid, RIF: rifampicin, S: Sensitive to specified antibiotic, R: Resistant to specified antibiotic.

a Strain similarity was assessed by tradition genotyping and was defined as being the same if the isolate pair shared < = 1 MIRU-VNTR locus difference between them.

b SNPs in coding regions are annotated using the reference amino acid, codon number and alternative amino acid. SNPs in non-coding regions (i.e. RNA genes and intergenic regions) are annotated using the reference nucleotide, gene coordinate and alternative nucleotide.

### Whole-genome sequence analysis identifies a high relapse rate among recurring TB episodes

Of 75 isolates obtained from the 36 *rc*TB cases, 65 (86.7%) had whole-genome sequences available for analysis. Ten genomes were not available due to poor gDNA yield (4) or poor average coverage of sequence reads (6).

**Figure 3:**
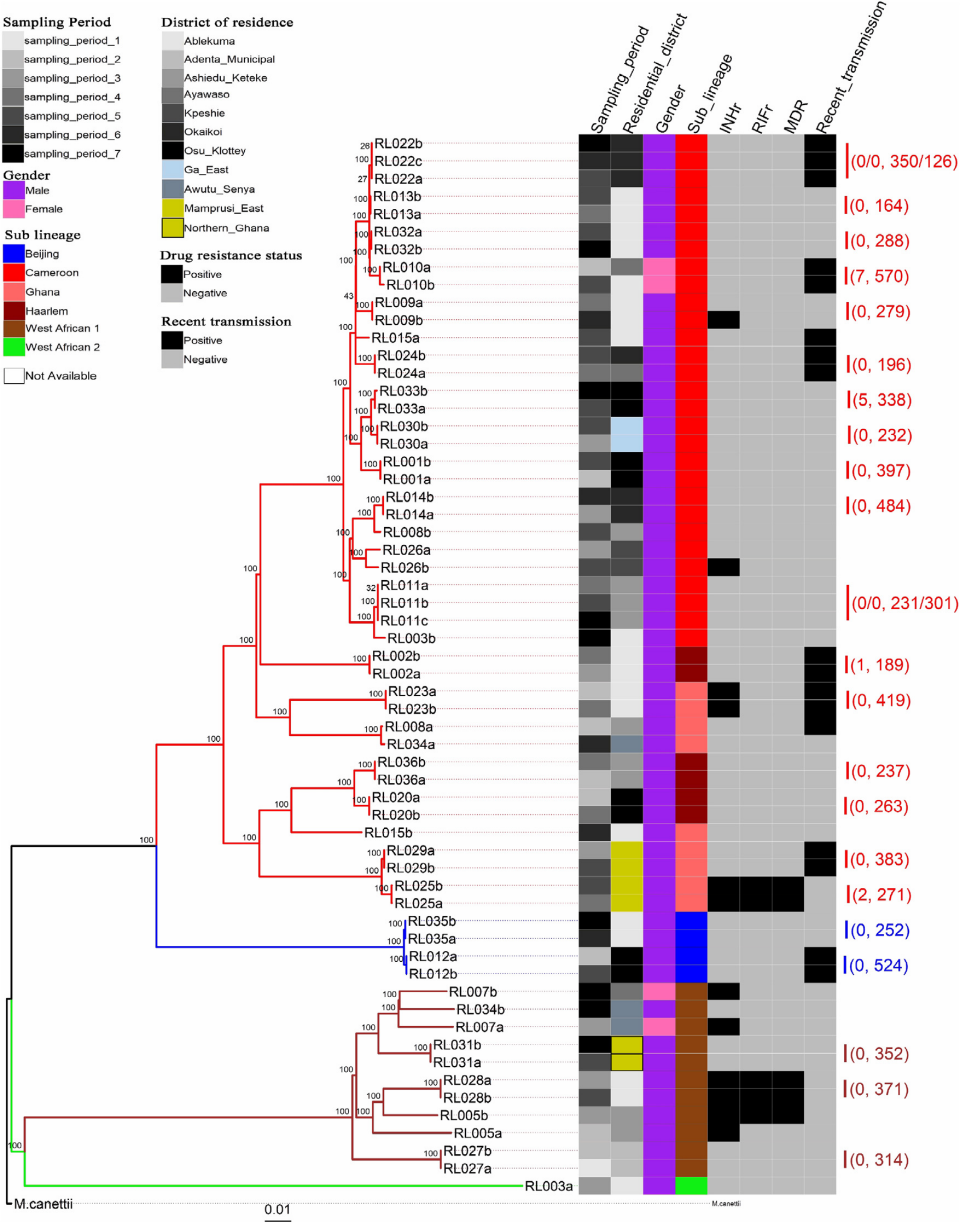
Phylogenetic reconstruction showing the genomic relationship between 60 *M. tuberculosis* complex isolates from 29 recurring TB cases. The tree was built with an alignment file containing 6485 variable positions. The heat map shows some characteristics of the cases, including sampling period (column 1), residential district (column 2), gender (column 3), sub-lineage (column 4), isoniazid resistance status (column 5), rifampicin resistance status (column 6), multi-drug resistance status (column 7) and whether that strain was involved in a recent transmission event (column 8). The color codes for the heat map are defined in the key. The 4 major branches constitute 4 MTBC lineages and are color-coded red for lineage 4, blue for lineage 2, brown for lineage 5 and green for lineage 6. Data to the right of the heat map shows the number of SNP differences and the number of days (in brackets) separating each isolate pair for relapse cases only. The tree was rooted with *Mycobacterium canettii*.

Based on our definitions for relapse and reinfection and available WGS data, *rc*TB was attributed to relapse in 61.1% (22/36) of participants and 19.4% (7/36) to reinfection. In the remaining 7 cases with no WGS data, MIRU-VNTR typing identified 5 as the same strain and 2 as different. Hence overall, there were 75.0% (27/36) relapse cases and 25.0% (9/36) reinfection cases. Of the 22 WGS confirmed relapse cases, 18 (81.8%) had no SNP (0 SNP) distance between their isolate pairs, with the remaining 4 separated by 1, 2, 5 and 7 SNPs, respectively (Table 1, Figure 3). Of the 9 reinfection cases, 1 participant (RL003) with HIV and diabetes mellitus was initially infected with a *Mycobacterium africanum* (MAF) strain and subsequently with an MTBss strain. Another participant (RL006) was initially infected with an MTBss strain and later with a MAF strain. The remaining 7 reinfection cases were infected with different strains, all belonging to the same MTBC lineage. The Cameroon sublineage was the most commonly associated with relapse (13/27, 48.1%), followed by the Ghana (5/27, 18.5%) and MAF West African I sublineages (3/27, 11.1%).

Statistical analysis comparing relapse vs reinfection cases showed no significant difference in the sociodemographic and clinical characteristics analyzed (Table 2). For instance, males with *rc*TB were equally as likely as females to be a relapse or re-infected case *(P* = 0.443). We also found no association of the cause of recurrence with age, marital status, formal education, other co-infections or sputum smear grade (*P* > 0.05).

**Table 2:** Demographic and clinical characteristics of participants with recurring TB episodes.

Characteristics	Relapse (N = 27) n(%)	Reinfection (N = 9) n(%)	Total (N = 36) n(%)	*P*-value
Gender (n = 36)				
Male	26 (96.3%)	8 (88.9%)	34 (94.4%)	0.443
Female	1 (3.7%)	1 (11.1%)	2 (5.6%)	
Age Category (n = 36)				
≤40	13 (48.1%)	6 (66.7%)	19 (52.8%)	0.451
>40	14 (51.9%)	3 (33.3%)	17 (47.2%)	
Time to recurring episode (n = 36)				
≤12 months	16 (59.3%)	5 (55.6%)	21 (58.3%)	1.000
>12 months	11 (40.7%)	4 (44.4%)	15 (41.7%)	
Marital Status (n = 33)				
Single	6 (24.0%)	3 (37.5%)	9 (27.7%)	0.521
Married	15 (60.0%)	5 (62.5%)	20 (60.6%)	
Others	4 (16.0%)	0 (0.0%)	4 (12.1%)	
Formal Education (at least lower grade) (n = 34)				
Yes	20 (80.0%)	8 (88.9%)	28 (82.4%)	1.000
No	5 (20.0%)	1 (11.1%)	6 (17.6%)	
HIV positive (n = 24)				
Yes	1 (5.9%)	1 (14.3%)	2 (8.3%)	0.683
No	16 (94.1%)	6 (85.7%)	22 (81.7%)	
Diabetes mellitus (n = 19)				
Yes	5 (35.7%)	2 (40.0%)	7 (36.8%)	1.000
No	9 (64.3%)	3 (60.0%)	12 (63.2%)	
Current smoker (n = 33)				
Yes	12 I48.0%)	3 (37.5%)	15 (45.4%)	0.699
No	13 (52.0%)	5 (62.5%)	18 (54.5%)	
Sputum smear grade (n = 36)				
≤1+	9 (33.3%)	3 (33.3%)	12 (33.3%)	1.000
>1+	18 (66.7%)	6 (66.7%)	24 (66.7%)	
Isoniazid resistant strain (n = 36)				
Yes	4 (14.8%)	3 (33.3%)	7 (19.4%)	0.333
No	23 (85.2%)	6 (66.7%)	29 (80.6%)	
Infecting MTBC lineage (n = 36)				
MTBss L2 and L4	23 (85.2%)	5 (55.6%)	28 (77.8%)	0.086
Maf L5 and L6	4 (14.8%)	4 (44.4%)	8 (22.2%)	

### Drug resistance profiles of recurring TB cases

Apart from 5 cases, all drug resistance profiles identified using either phenotypic means or by MTBDRplus were identical to that suggested by the WGS analysis for drug-resistant mutations. At least 12 isolates had 1 form of drug resistance, either INH resistance only (7) or MDR (5). Half of these resistant isolates belonged to the MAF lineage (5 L5 and 1 L6), followed by the L4 Ghana (4) and L4 Cameroon (2) sublineages. In addition to INH and RIF resistance, some isolates also had resistance-conferring mutations to other antibiotics, including streptomycin (RL005b, RL026), pyrazinamide (RL016b, RL025, RL026 and RL028) and ethambutol (RL006a, RL016b and RL025) (Supplementary Table 2).

Isolates from participant RL005 had a discrepant drug resistance profile, whereas the MTBDRplus suggested INH resistance (katG MUT 1 Present) for the primary episode (RL005a), no resistance-associated mutation was found in WGS analysis (Table 1, Supplementary Table 2). We observed both INH and RIF resistance in the isolate from the secondary episode (rpoBMUT2B present, katG WT absent, katG MUT1 present) using MTBDR plus, but WGS analysis confirmed only INH resistance. RL007 also had discrepant drug resistance profile results. Except for *rc*TB cases with different strains in both episodes, all other *rc*TB cases had the same set of non-resistant associated mutations in the list of resistant genes investigated (Supplementary Table 3).

### Characteristics of individuals belonging to household-related TB transmission cases

A total of 26 isolates from 11 households were analyzed for household-related transmission. The majority of the cases were male (17/26, 65.4%) with a median age of 40 (IQR, 26-49); the remaining 9/26 (34.6%) were female with a median age of 27 (IQR, 20-30). Of the 26 participants, 19 (73.1%) had a smear grade >1+. Of the participants tested for co-infection, only 1 tested positive for diabetes mellitus and none for HIV.

Of the 26 isolates, 21 (70.8%) had whole-genome sequences available for analysis. Five genomes were excluded; 3 due to poor gDNA yield and 1 each due to failed library and possible mixed-infection/contamination (Figure 2). Of the 11 (72.7%) household cases, 8 had individuals infected with the same strain, 2 had individuals infected with different MTBC strains, and 1 (FT014) had 2 participants with the same MIRU-VNTR allelic pattern but separated by a genomic distance of 35 SNPs. All participants had MTBss lineages, with the predominant sublineage being Cameroon (46.2%, 12/26), followed by Ghana (19.2%, 5/26) and Beijing (15.4%, 4/26).

For all cases with WGS data, the drug resistance profile was the same for both phenotypic and WGS mutation-predicted resistance. Only 11.5% (3/26) of participants had an INH-resistant strain, and none had a RIF-resistant strain (Table 3). Two of the 3 INH-resistant cases were female, infected with the Ghana sublineage. Some isolates also had resistance-associated mutations to other anti-TB drugs, including streptomycin (FT007, FT014a), ethambutol (FT001 and FT016) and capreomycin (FT001 and FT016) (Supplementary Table 2).

**Table 3:** Characteristics of cases involved in household-related transmission.

Participant ID	Gender	Age (yrs)	Smear grade	MTBC lineage/Spoligotype	Strain similarity		Drug resistance			Pairwise SNP difference	WGS status
					Traditional genotyping^a^	WGS	INH	RIF	Mutation identified^b^		
FT001a	Male	26	3+	Ghana	Same	Same	S	S		0	Available
FT001b	Female	20	2+	Ghana	Same	Same	S	S			Available
FT002a	Female	28	3+	Cameroon	Similar	NA	S	S			NA
FT002b	Male	29	scanty	Cameroon	Similar	NA	S	S			Available
FT003a	Male	19	3+	Cameroon	Same	Same	S	S		0	Available
FT003b	Female	50	3+	Cameroon	Same	Same	S	S			Available
FT004a	Male	15	3+	Cameroon	Same	Same	S	S		2	Available
FT004b	Male	49	1+	Cameroon	Same	Same	S	S			Available
FT004c	Male	27	2+	Cameroon	Same	NA	S	S			NA
FT006a	Male	NA	3+	Delhi/CAS	Different	NA	R	S	−8T > A,fabG1, Rv1483		Available
FT006b	Male	13	3+	Cameroon	Different	NA	S	S			NA
FT007a	Female	45	2+	Beijing	Similar	Same	S	S		0	Available
FT007b	Female	16	3+	Beijing	Similar	Same	S	S			Available
FT012a	Male	49	2+	Haarlem	Same	Same	S	S		0/0/0	Available
FT012b	Male	49	scanty	Haarlem	Same	Same	S	S			Available
FT012c	Male	50	2+	Haarlem	Similar	Same	S	S			Available
FT012d	Male	54	3+	S	Different	NA	S	S			NA
FT013a	Male	40	3+	Cameroon	Same	Same	S	S		0	Available
FT013b	Male		3+	Cameroon	Same	Same	S	S			Available
FT014a	Male	28	3+	Beijing	Same	Similar	S	S		35	Available
FT014b	Male	40	1+	Beijing	Same	Similar	S	S			Available
FT015a	Female	15	scanty	Cameroon	Same	Same	S	S		0	Available
FT015b	Male	44	1+	Cameroon	Same	Same	S	S			Available
FT016a	Female	27	scanty	Ghana	Same	Same	R	S	−15C > T,fabGI,Rv1483	4	Available
FT016b	Female	30	3+	Ghana	Same	Same	R	S	−15C > T,fabGI,Rv1483		Available
FT016c	Female	24	2+	Ghana	Same	NA	S	S			NA

There were 4 participants in household FT012 and 3 participants in each of FT004 and FT016.

NA: not available, WGS: whole-genome sequence, SNP: single nucleotide polymorphism, INH: isoniazid, RIF: rifampicin, S: sensitive to specified antibiotic, R: resistant to specified antibiotic.

a Traditional genotyping clusters are defined as previously described clusters using mycobacterial interspersed repetitive-unit-variable number tandem-repeat analysis and spoligotyping genotyping tools.

b SNPs in coding regions are annotated using the reference amino acid, codon number and alternative amino acid. SNPs in non-coding regions (i.e. RNA genes and intergenic regions) are annotated using the reference nucleotide, gene coordinate and alternative nucleotide.

### Evidence of recent household transmission

**Figure 4:**
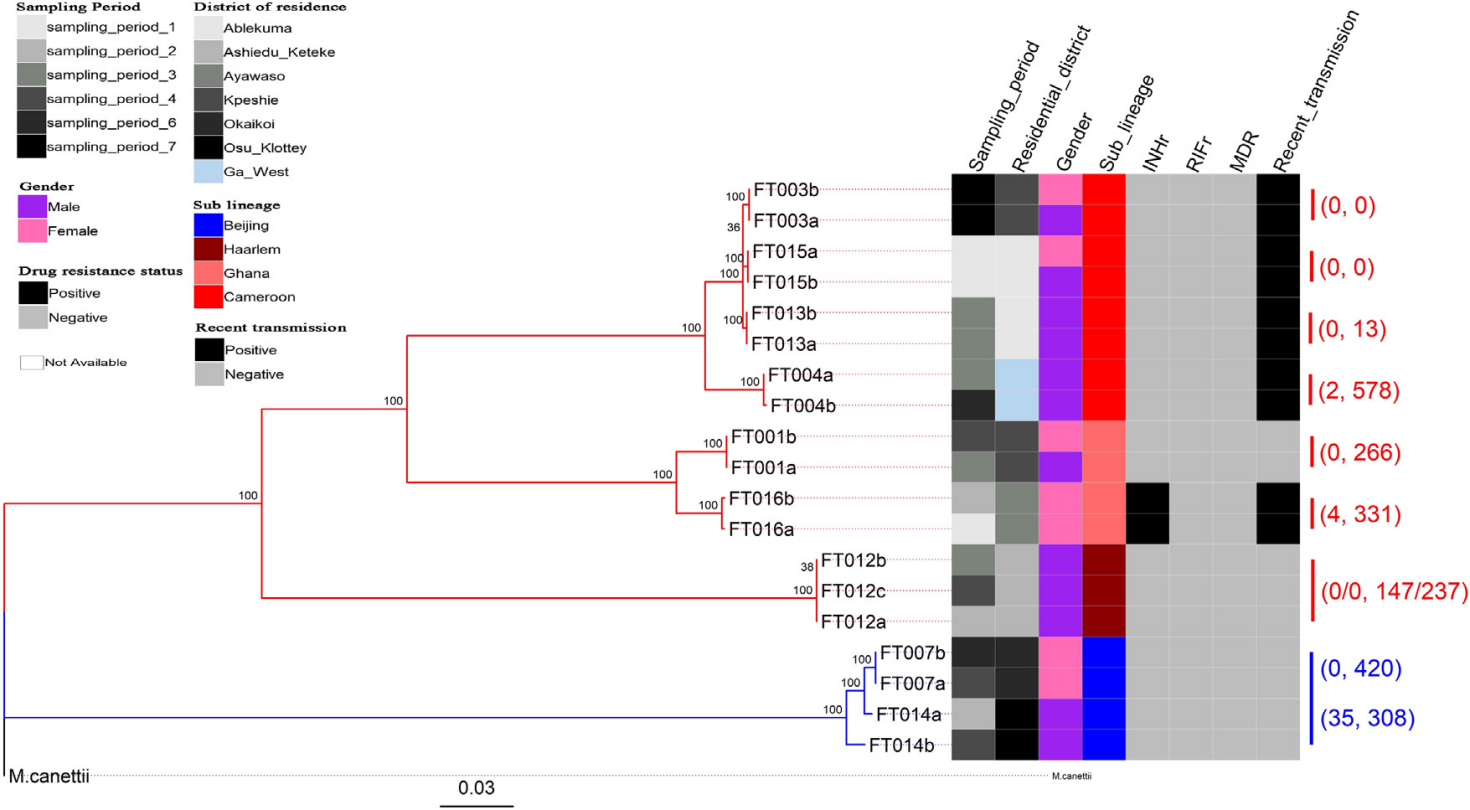
Phylogenetic reconstruction showing the genomic relationship between 19 *M. tuberculosis* complex isolates from 9 household transmission groups. The tree was built with an alignment file containing 2022 variable positions. The heat map shows some characteristics of the cases, including sampling period (column 1), residential district (column 2), gender (column 3), sub-lineage (column 4), isoniazid resistance status (column 5), rifampicin resistance status (column 6), multi-drug resistance status (column 7) and involvement of strain in a recent transmission event (column 8). The color codes for the heat map are defined in the key. The 2 major branches constitute 2 MTBC lineages and color-coded blue for lineage 2, red for lineage 4. Data to the right of the heat map shows the number of SNP differences and the number of days (in brackets) separating each isolate pair. The tree was rooted with *M. canettii*.

**Figure 5:**
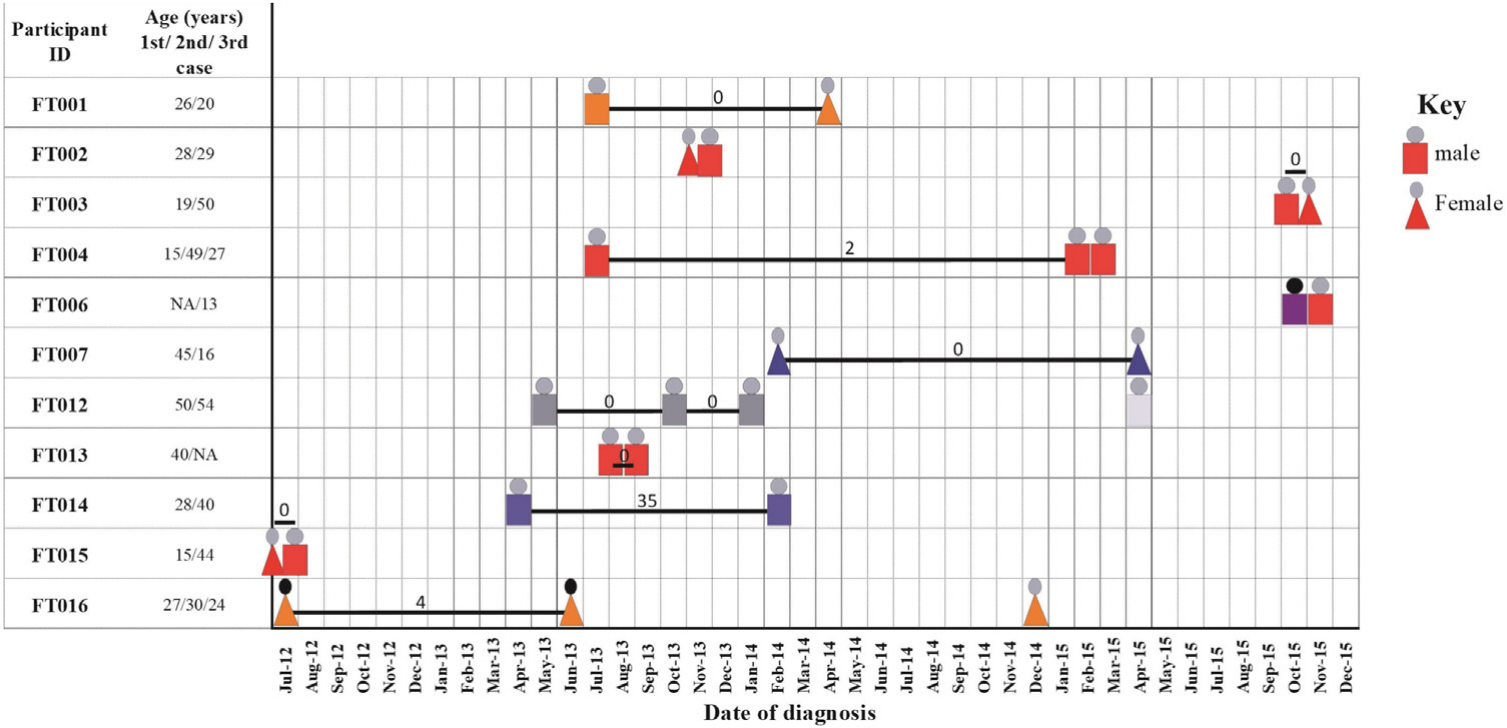
Time until the event of household-related tuberculosis cases. The time of diagnosis for each household-related TB case is shown. The color codes represent the various human-adapted *M. tuberculosis* complex *sub-*lineages; red for ‘Cameroon’, orange for ‘Ghana’, blue for ‘Beijing’, Grey for ‘Haarlem’ and light grey for ‘S’ sublineages. For household-related cases that are likely the same or similar MTBC strain, the SNP distances between each pair are indicated on the bars.

Of the 26 participants, 11 reported contact with more than 1 TB patient, 9 from a family member or individual living in the same house who had been coughing within 1 year, and 2 from close workmates. FT004, a Cameroon sublineage case, involved 3 male participants in the same household. Participant FT004a (aged 15 years) was the first case in this household to be reported, he came into contact with a TB patient at his workplace 1 year ago. He shared 2 SNP distance with the next case (FT004b), being his father (aged 49). The 2 SNP distance was accumulated within 578 days (Figure 4, Figure 5). The third case (FT004c) had no WGS data but had the same MIRU-VNTR allelic pattern as the 2 previous cases. Participants FT004b and FT004c shared the same room in the house. Participant FT007b, a 16-year-old girl, had also been in contact with her mother (FT007a, Beijing sub-lineage, aged 45) within 420 days and was infected with the same strain (0 SNP distance).

## 15 -locus MIRU-VNTR typing is sufficient to predict the cause of recurring TB and identify suspected household-related TB transmission

**Figure 6:**
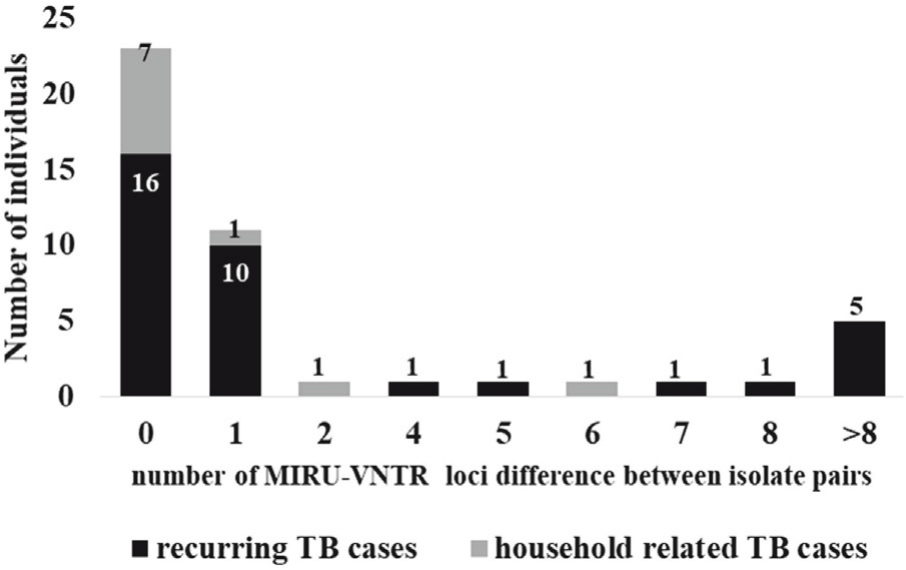
Number of MIRU-VNTR allelic variations between identified recurrent TB cases (black bars) and household-related cases (grey bars).

**Figure 7:**
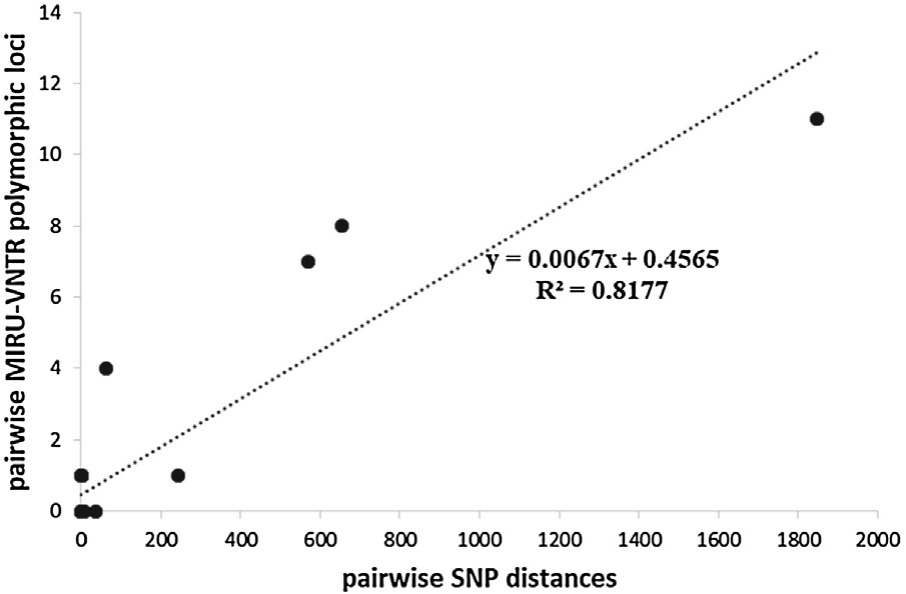
Concordance between MIRU-VNTR allelic polymorphism and WGS SNP distances.

MIRU-VNTR analysis revealed that 75.6% (34/45) paired isolates with MIRU-VNTR allelic information had < = 1 locus variance between isolate pairs (Figure 6, Supplementary Table 3). There was a high concordance (94.6%, Kappa = 0.7702, *P* < 0.001) and positive correlation (R^2^ = 0.817, *P* < 0.001, Figure 7) between 15-locus MIRU-VNTR typing and WGS typing. MIRU-VNTR and WGS found all other isolate pairs to be the same or similar, with < = 7 SNPs separating each isolate pair, with the exception of 1 isolate pair (FT014) which MIRU-VNTR predicted to be the same but WGS found 35 SNP separation. All remaining cases that MIRU-15 predicted as different strains were confirmed by WGS with >62 SNP separation.

## Discussion

This study aimed to delineate the occurrence of recurring TB and describe household-related transmission among individuals with pulmonary TB reporting to selected health facilities in Ghana, by combining WGS and standard molecular epidemiological tools. Key findings from our analysis are: 1) the majority (75.0%, 27/36) of recurring TB cases result from relapse of the previous infection; 2) household-related TB cases are likely the result of recent TB transmission within the house or from neighboring recent transmission events; 3) although WGS is ideal, 15-locus MIRU-VNTR typing is sufficient to predict the cause of TB recurrence and is also able to predict recent household-related transmission.

Studies in India by Velayutham and colleagues have reported that up to 55% of patients had TB recurrence within 3 months post treatment, and 77% within 6 months (Velayutham et al., 2018). The majority (58.3%, 21/36) of TB recurrences occurred within the first year post treatment, similar to observations made elsewhere (Thomas et al., 2005, Velayutham et al., 2018). It is assumed that in high endemic regions, *rc*TB cases generally result from reinfection rather than relapse (Parvaresh et al., 2018, van Rie et al., 1999). However, studies conducted in endemic regions such as India and China have attributed up to 93% of *rc*TB cases to relapse (Velayutham et al., 2018, Zong et al., 2018). However, these studies used MIRU-VNTR to discriminate between strains which may overestimate the true incidence of relapse among *rc*TB cases. However, these observations were similar to our finding (which is strengthened by the use of WGS) that up to 75% of *rc*TB cases may result from relapse.

The predominance of relapse over reinfection indicates high-quality public health practices and a low risk of local transmission. However, relapse cases have been associated with MDR development (Alipanah et al., 2018, Yang et al., 2017). Measures to reduce relapse cases and improve treatment outcome include adherence interventions such as patient education and counseling, psychological interventions, incentives and enablers, and digital health technologies (Alipanah et al., 2018, Amo-Adjei and Awusabo-Asare, 2013). These measures are necessary as drug-resistant strains can emerge due to a lack of treatment adherence.

An increase in Ghana's TB treatment success has been reported, from 44% in 1997 to 87% in 2013, with current rates estimated at 85% (Amo-Adjei and Awusabo-Asare, 2013, WHO, 2018b). With this high treatment success, we expected that *rc*TB cases would be due to reinfection with a new strain rather than relapse; however, we observed the contrary. Therefore we intend to explore further the drug resistance profiles of isolates from our study cohort. Of the 4 relapse cases (RL005, RL018, RL025 and, RL028) with MDR-TB strains during their secondary episode, 2 were confirmed cured after their primary episode. One participant (RL028) with an MDR-TB strain at primary episode was confirmed cured and returned to his community. After 1 year this individual relapsed with the same strain and may potentially have spread the MDR bacilli, which have a propensity to transmit, to other people (Cohen et al., 2019, Lalor et al., 2018). As part of a good control system, public health measures, including contact tracing, are needed to control the spread of such difficult-to-treat MDR-TB strains.

In addition to *rc*TB cases, our analysis of household-related TB cases identified that 8/11 (72.7%) households were involved in recent TB transmission. We acknowledge that for household-related transmission studies, the classical approach would be to adopt a contact tracing method. Though we did not use this approach, we took advantage of our population-based study spanning a large enough time period to capture such cases. We showed that most household-related TB cases are due to recent transmission of the same strain, and >80% of the first identified case in each household had a smear grade of >1+, implying high infectiousness. Although we did not identify any MDR strain in these cases, strains can evolve into MDR strains, as we identified in the recurrent cohort. MDR transmission can hinder TB control locally and internationally (Cohen et al., 2019, Lalor et al., 2018), we therefore recommend that contact tracing, compound house screening and follow-up study be employed to help identify household-related cases early enough to control the spread of the disease.

Some research groups have considered if 15-locus MIRU-VNTR is sufficient to study strain relationships (Gibson et al., 2005, Kozinska and Augustynowicz-Kopec, 2016). Here, we show that although WGS performs best, 15-locus MIRU-VNTR typing is sufficient to predict the cause of TB recurrence and household-related transmission. Hence, we recommend 15-locus MIRU-VNTR typing as an initial screening tool in resource-limited settings to improve TB control through early identification of infection source and inform treatment selection based on the previous antimicrobial susceptibility of that strain.

To the best of our knowledge, this is the first extensive report of analysis of recurring TB cases and household-related TB transmission using WGS in Ghana and West Africa. However, our study was limited by our 3.5 years for participant recruitment which meant that we did not obtain isolates from later recurring TB cases, and we did not have isolates for recurring cases with a primary episode occurring before the study period. Consequently, we had a lower than expected number of cases. Another limitation is that we had no concrete previous treatment outcome data for half (18/36) of the *rc*TB cases, potentially leading to inaccurate reporting of the relapse prevalence. However, this does not override our observation that most *rc*TB cases result from relapse, given that 82% (9/11) of individuals initially declared cured became relapse cases.

## Conclusion

It is possible to monitor recurring TB cases and follow-up household-related transmission in a resource-limited setting. We recommend that local control programs invest more resources into such studies as they have been proven to provide vital findings that positively influence TB control (Velayutham et al., 2018).

## Funding

This work was supported by a Wellcome Trust Intermediate Fellowship Grant (097134/Z/11/Z) to Dorothy Yeboah-Manu. Funders had no role in the study design, collection, analysis and interpretation of data, in the writing of the report, nor in the decision to submit the paper for publication. DYM, PA and IDO had full access to all the data used in the study. The corresponding author had the final responsibility for the decision to submit for publication.

## Ethical approval

The Scientific and Technical Committee and the Institutional Review Board of the Noguchi Memorial Institute for Medical Research (NMIMR), University of Ghana (FWA00001824) reviewed and approved all protocols and procedures for this study.

## Declaration of interest

We declare that we have no conflict of interest.
