# Supplementary material for: Genomic epidemiological analysis identifies high relapse among individuals with recurring tuberculosis and provides evidence of recent household-related transmission of tuberculosis in Ghana
Source: Int J Infect Dis. 2021 May;106:13–22. doi: 10.1016/j.ijid.2021.02.110 (PMC8134059; doi:10.1016/j.ijid.2021.02.110)
Supplement: Supplementary file 1 [file mmc1.pdf]

SUPPLEMENTARY MATERIAL

Genomic epidemiological analysis identifies high relapse among individuals with recurring tuberculosis and provides evidence of household recent TB transmission in Ghana

---

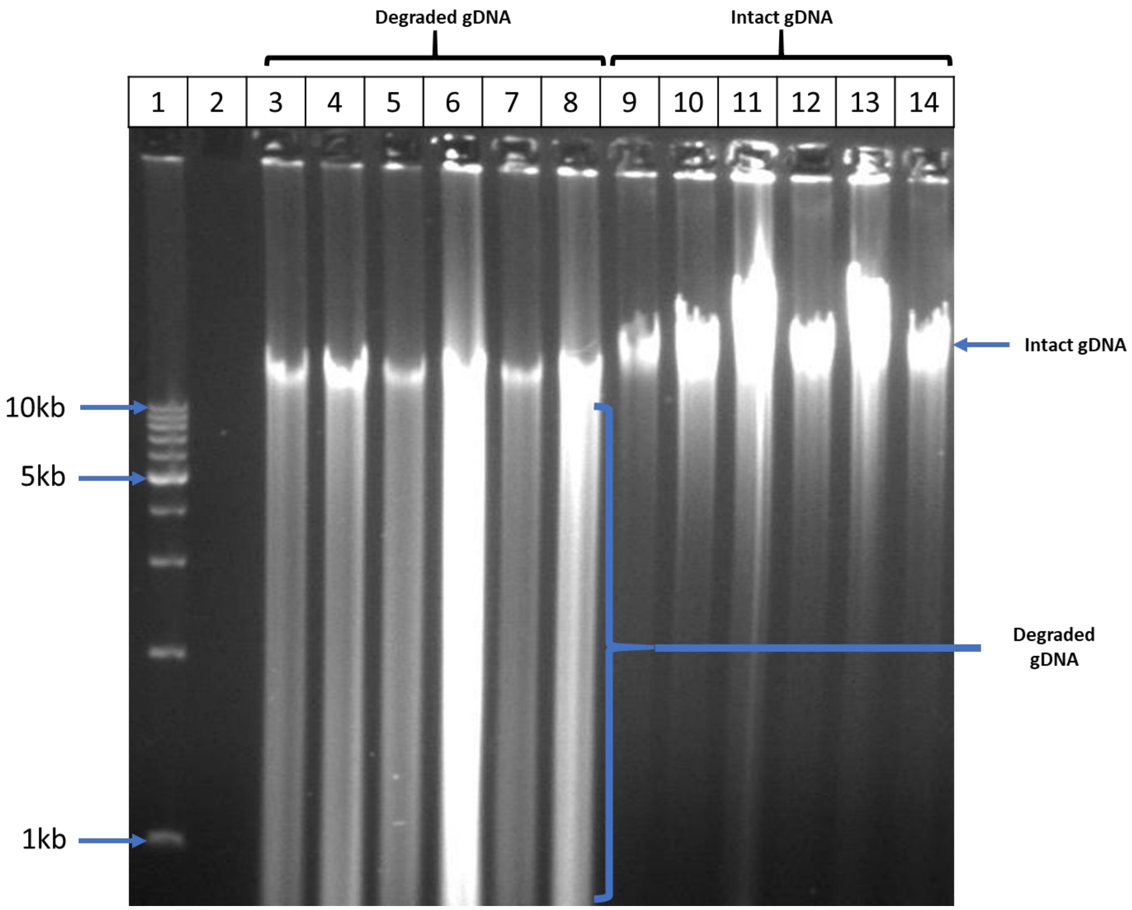

Supplementary figure 1. Gel electrophoretic assessment of modified CTAB protocol. The gel shows the electrophoretic mobility pattern of extracted genomic DNA (gDNA) comparing the two protocols run alongside a 1kb molecular weight marker (lane 1) and an extraction control (lane 2). Lanes 3 to 8 contains gDNA obtained using the previous protocol (Bacterial cells heat inactivated at 95 ºC for 1hour), lanes 9 to 14 contains gDNA obtained using the modified protocol (Bacterial cells heat inactivated at 80 ºC for 30mins). The previous protocol results in degraded DNA whereas the modified protocol produces intact gDNA bands.

Supplementary table 1. Availability of raw sequence reads

Genome number	Isolate ID	Transmission code	Isolation institution	Sequencing institution	Provider	Accession number	Country of origin
G04165	TBNm 2263	RL005b	NMIMR	WTSI	DYM	ERR1215476	Ghana
G08474	TBNm 3323	RL031a	NMIMR	WTSI	DYM	ERR1082123	Ghana
G08479	TBNm 1672	FU031a	NMIMR	WTSI	DYM	ERR1082128	Ghana
G08481	TBNm 1821	RL003a	NMIMR	WTSI	DYM	ERR1082130	Ghana
G08497	TBNm 2359	RL007a	NMIMR	WTSI	DYM	ERR1203055	Ghana
G08663	TBNm 1615	RL005a	NMIMR	WTSI	DYM	ERR702410	Ghana
G08714	TBNm 1803B	RL028b	NMIMR	WTSI	DYM	ERR751314	Ghana
G08729	TBNm 1010	RL027a	NMIMR	WTSI	DYM	ERR751333	Ghana
G26737	TBNm 2399	RL002a	NMIMR	STPH	DYM	SRR11444212	Ghana
G26741	TBNm 1541	RL012a	NMIMR	STPH	DYM	SRR11461691	Ghana
G26745	TBNm 4197	FT003a	NMIMR	STPH	DYM	SRR11444412	Ghana
G26748	TBNm 2563	FT014b	NMIMR	STPH	DYM	SRR11461685	Ghana
G26760	TBNm 1541B	RL012b	NMIMR	STPH	DYM	SRR11461680	Ghana
G26762	TBNm 3823	FT007b	NMIMR	STPH	DYM	SRR11461660	Ghana
G26776	TBNm 2722	RL020b	NMIMR	STPH	DYM	SRR11461681	Ghana
G26797	TBNm 2070	RL029a	NMIMR	STPH	DYM	SRR11444249	Ghana
G26798	TBNm 3295	RL015a	NMIMR	STPH	DYM	SRR11444093	Ghana
G26825	TBNm 3415B	RL033b	NMIMR	STPH	DYM	SRR11461667	Ghana
G26831	TBNm 2428B	RL025b	NMIMR	STPH	DYM	SRR11461690	Ghana
G26832	TBNm 3378B	RL032b	NMIMR	STPH	DYM	SRR11461670	Ghana
G26854	TBNm 2558	RL024a	NMIMR	STPH	DYM	SRR11461686	Ghana
G26855	TBNm 2772	RL023b	NMIMR	STPH	DYM	SRR11461678	Ghana
G26870	TBNm 1809	FT001a	NMIMR	STPH	DYM	SRR11461701	Ghana
G26871	TBNm 2070B	RL029b	NMIMR	STPH	DYM	SRR11461699	Ghana
G26872	TBNm 2598	FT007a	NMIMR	STPH	DYM	SRR11461682	Ghana
G26874	TBNm 2304B	RL030b	NMIMR	STPH	DYM	SRR11461693	Ghana
G26875	TBNm 4046	RL003b	NMIMR	STPH	DYM	SRR11461657	Ghana
G26878	TBNm 3755	RL009b	NMIMR	STPH	DYM	SRR11461661	Ghana
G26895	TBNm 3209	RL011b	NMIMR	STPH	DYM	SRR11461674	Ghana
G26896	TBNm 2981	RL002b	NMIMR	STPH	DYM	SRR11461677	Ghana
G32002	TBNm 1797	FT004a	NMIMR	STPH	DYM	SRR11444291	Ghana
G32005	TBNm 1716B	RL008b	NMIMR	STPH	DYM	SRR11461652	Ghana
G32006	TBNm 2749	FT001b	NMIMR	STPH	DYM	SRR11461679	Ghana
G32009	TBNm 2502	RL011a	NMIMR	STPH	DYM	SRR11461688	Ghana
G32010	TBNm 3623	FT004b	NMIMR	STPH	DYM	SRR11444062	Ghana
G32753	TBNm 2428	RL025a	NMIMR	STPH	DYM	SRR11444205	Ghana
G32756	TBNm 1939	FT013a	NMIMR	STPH	DYM	SRR11444265	Ghana

Genome number	Isolate ID	Transmission code	Isolation institution	Sequencing institution	Provider	Accession number	Country of origin
G32762	TBNm 3672	RL035a	NMIMR	STPH	DYM	SRR11461663	Ghana
G32767	TBNm 1673	FT012a	NMIMR	STPH	DYM	SRR11461658	Ghana
G32783	TBNm 3416	RL026b	NMIMR	STPH	DYM	SRR11461666	Ghana
G32784	TBNm 1468	RL023a	NMIMR	STPH	DYM	SRR11444351	Ghana
G32785	TBNm 3245	RL010b	NMIMR	STPH	DYM	SRR11461673	Ghana
G32786	TBNm 2442	FT012c	NMIMR	STPH	DYM	SRR11461689	Ghana
G32792	TBNm 1995	FT013b	NMIMR	STPH	DYM	SRR11461700	Ghana
G32798	TBNm 2407	RL026a	NMIMR	STPH	DYM	SRR11461692	Ghana
G32810	TBNm 3111	RL009a	NMIMR	STPH	DYM	SRR11461675	Ghana
G32817	TBNm 1780	RL020a	NMIMR	STPH	DYM	SRR11461651	Ghana
G32836	TBNm 2984	RL001b	NMIMR	STPH	DYM	SRR11461676	Ghana
G32847	TBNm 1502	RL010a	NMIMR	STPH	DYM	SRR11444348	Ghana
G32854	TBNm 1547	FU049a	NMIMR	STPH	DYM	SRR11444343	Ghana
G32857	TBNm 2572	RL013a	NMIMR	STPH	DYM	SRR11461684	Ghana
G32864	TBNm 4045	RL022b	NMIMR	STPH	DYM	SRR11461659	Ghana
G32865	TBNm 2138B	RL014b	NMIMR	STPH	DYM	SRR11461697	Ghana
G32866	TBNm 3378	RL032a	NMIMR	STPH	DYM	SRR11461671	Ghana
G32882	TBNm 1701	RL001a	NMIMR	STPH	DYM	SRR11461653	Ghana
G32966	TBNm 4199	FT003b	NMIMR	STPH	DYM	SRR11444411	Ghana
G32974	TBNm 4289	RL015b	NMIMR	STPH	DYM	SRR11461654	Ghana
G32979	TBNm 4112	RL007b	NMIMR	STPH	DYM	SRR11461656	Ghana
G32993	TBNm 2502B	RL011c	NMIMR	STPH	DYM	SRR11461687	Ghana
G33016	TBNm 2939	FU080a	NMIMR	STPH	DYM	SRR11444129	Ghana
G33026	TBNm 2166	FT012b	NMIMR	STPH	DYM	SRR11461696	Ghana
G33036	TBNm 1028	FT015a	NMIMR	STPH	DYM	SRR11444358	Ghana
G33048	TBNm 3575B	RL034b	NMIMR	STPH	DYM	SRR11461664	Ghana
G33063	TBNm 2572B	RL013b	NMIMR	STPH	DYM	SRR11461683	Ghana
G33065	TBNm 1716	RL008a	NMIMR	STPH	DYM	SRR11444305	Ghana
G33066	TBNm 1018	FT016a	NMIMR	STPH	DYM	SRR11444091	Ghana
G33068	TBNm 1034	FT015b	NMIMR	STPH	DYM	SRR11444325	Ghana
G33081	TBNm 3323B	RL031b	NMIMR	STPH	DYM	SRR11461672	Ghana
G33095	TBNm 1547B	FU049b	NMIMR	STPH	DYM	SRR11461669	Ghana
G33097	TBNm 3125	FU080b	NMIMR	STPH	DYM	SRR11444109	Ghana
G33098	TBNm 2304	RL030a	NMIMR	STPH	DYM	SRR11461694	Ghana
G33105	TBNm 3575	RL034a	NMIMR	STPH	DYM	SRR11461665	Ghana
G33128	TBNm 1538	FT014a	NMIMR	STPH	DYM	SRR11461702	Ghana
G33131	TBNm 1803	RL028a	NMIMR	STPH	DYM	SRR11461650	Ghana
G33138	TBNm 3672B	RL035b	NMIMR	STPH	DYM	SRR11461662	Ghana
G33139	TBNm 2238	FU031b	NMIMR	STPH	DYM	SRR11461695	Ghana

Genome number	Isolate ID	Transmission code	Isolation institution	Sequencing institution	Provider	Accession number	Country of origin
G33140	TBNm 3060	RL022a	NMIMR	STPH	DYM	SRR11444116	Ghana
G33150	TBNm 3415	RL033a	NMIMR	STPH	DYM	SRR11461668	Ghana
G33157	TBNm 3161	RL024b	NMIMR	STPH	DYM	SRR11444104	Ghana
G33160	TBNm 1010B	RL027b	NMIMR	STPH	DYM	SRR11461703	Ghana
G33163	TBNm 1694	FT016b	NMIMR	STPH	DYM	SRR11444311	Ghana
G33166	TBNm 4277	RL022c	NMIMR	STPH	DYM	SRR11461655	Ghana
G33167	TBNm 2138	RL014a	NMIMR	STPH	DYM	SRR11461698	Ghana

NA = Accession numbers are not yet available; will be updated when they are available

NB. Genomes of FT012a and FT012c are same for RL036a and RL036b respectively as indicated in methods. The median genome coverage was 68x.

Supplementary table 2. List of other mutations identified among drug resistant associated genes**.**

Participant_ID	Chromosome_Position	Gene_locus	Gene	Mutation	Estimated_fraction	Drug_resistant_to
FT001a	1918647	Rv1694	tlyA	Asn236Lys	0.973	capreomycin
FT001a	4247451	Rv3795	embB	Ala313Val	0.988	ethambutol
FT001a	7362	Rv0006	gyrA	Glu21Gln	1	non-resistant associated
FT001a	7585	Rv0006	gyrA	Ser95Thr	1	non-resistant associated
FT001a	9304	Rv0006	gyrA	Gly668Asp	1	non-resistant associated
FT001a	759604	Rv0667	rpoB	-203A>G	0.973	non-resistant associated
FT001a	781395	Rv0682	rpsL	-165T>C	1	non-resistant associated
FT001a	1917972	Rv1694	tlyA	33A>G	1	non-resistant associated
FT001a	2746995	Rv2447c	folC	Val202Ile	0.957	non-resistant associated
FT001a	4242643	Rv3793	embC	2781C>T	1	non-resistant associated
FT001b	1918647	Rv1694	tlyA	Asn236Lys	1	capreomycin
FT001b	4247451	Rv3795	embB	Ala313Val	1	ethambutol
FT001b	7362	Rv0006	gyrA	Glu21Gln	1	non-resistant associated
FT001b	7585	Rv0006	gyrA	Ser95Thr	1	non-resistant associated
FT001b	9304	Rv0006	gyrA	Gly668Asp	1	non-resistant associated
FT001b	759604	Rv0667	rpoB	-203A>G	1	non-resistant associated
FT001b	781395	Rv0682	rpsL	-165T>C	1	non-resistant associated
FT001b	1917972	Rv1694	tlyA	33A>G	1	non-resistant associated
FT001b	2746995	Rv2447c	folC	Val202Ile	0.985	non-resistant associated
FT001b	4242643	Rv3793	embC	2781C>T	1	non-resistant associated
FT003a	7362	Rv0006	gyrA	Glu21Gln	1	non-resistant associated
FT003a	7585	Rv0006	gyrA	Ser95Thr	1	non-resistant associated
FT003a	9304	Rv0006	gyrA	Gly668Asp	0.989	non-resistant associated
FT003a	781395	Rv0682	rpsL	-165T>C	1	non-resistant associated
FT003a	1917972	Rv1694	tlyA	33A>G	1	non-resistant associated

Participant_ID	Chromosome_Position	Gene_locus	Gene	Mutation	Estimated_fraction	Drug_resistant_to
FT003a	2289037	Rv2043c	pncA	Pro69Ser	1	non-resistant associated
FT003a	4242550	Rv3793	embC	2688C>G	1	non-resistant associated
FT003a	4242643	Rv3793	embC	2781C>T	1	non-resistant associated
FT003a	4326739	Rv3854c	ethA	735G>C	1	non-resistant associated
FT003a	4328004	Rv3855	ethR	456G>A	0.987	non-resistant associated
FT003b	7362	Rv0006	gyrA	Glu21Gln	1	non-resistant associated
FT003b	7585	Rv0006	gyrA	Ser95Thr	1	non-resistant associated
FT003b	9304	Rv0006	gyrA	Gly668Asp	1	non-resistant associated
FT003b	781395	Rv0682	rpsL	-165T>C	1	non-resistant associated
FT003b	1917972	Rv1694	tlyA	33A>G	1	non-resistant associated
FT003b	2289037	Rv2043c	pncA	Pro69Ser	1	non-resistant associated
FT003b	4242550	Rv3793	embC	2688C>G	1	non-resistant associated
FT003b	4242643	Rv3793	embC	2781C>T	1	non-resistant associated
FT003b	4326739	Rv3854c	ethA	735G>C	1	non-resistant associated
FT003b	4328004	Rv3855	ethR	456G>A	1	non-resistant associated
FT004a	7362	Rv0006	gyrA	Glu21Gln	1	non-resistant associated
FT004a	7585	Rv0006	gyrA	Ser95Thr	1	non-resistant associated
FT004a	9304	Rv0006	gyrA	Gly668Asp	1	non-resistant associated
FT004a	781395	Rv0682	rpsL	-165T>C	1	non-resistant associated
FT004a	1917972	Rv1694	tlyA	33A>G	1	non-resistant associated
FT004a	4242550	Rv3793	embC	2688C>G	0.957	non-resistant associated
FT004a	4242643	Rv3793	embC	2781C>T	1	non-resistant associated
FT004a	4326739	Rv3854c	ethA	735G>C	1	non-resistant associated
FT004a	4328004	Rv3855	ethR	456G>A	1	non-resistant associated
FT004b	7362	Rv0006	gyrA	Glu21Gln	1	non-resistant associated
FT004b	7585	Rv0006	gyrA	Ser95Thr	1	non-resistant associated
FT004b	9304	Rv0006	gyrA	Gly668Asp	1	non-resistant associated
FT004b	781395	Rv0682	rpsL	-165T>C	0.963	non-resistant associated
FT004b	1917972	Rv1694	tlyA	33A>G	1	non-resistant associated

Participant_ID	Chromosome_Position	Gene_locus	Gene	Mutation	Estimated_fraction	Drug_resistant_to
FT004b	4242550	Rv3793	embC	2688C>G	0.923	non-resistant associated
FT004b	4242643	Rv3793	embC	2781C>T	1	non-resistant associated
FT004b	4326739	Rv3854c	ethA	735G>C	0.962	non-resistant associated
FT004b	4328004	Rv3855	ethR	456G>A	0.971	non-resistant associated
FT006a	7362	Rv0006	gyrA	Glu21Gln	0.988	non-resistant associated
FT006a	7585	Rv0006	gyrA	Ser95Thr	1	non-resistant associated
FT006a	9304	Rv0006	gyrA	Gly668Asp	0.981	non-resistant associated
FT006a	759746	Rv0667	rpoB	-61C>T	1	non-resistant associated
FT006a	761279	Rv0667	rpoB	Ile491Met	1	non-resistant associated
FT006a	762434	Rv0667	rpoB	2628T>G	1	non-resistant associated
FT006a	763031	Rv0667	rpoB	3225T>C	1	non-resistant associated
FT006a	781395	Rv0682	rpsL	-165T>C	1	non-resistant associated
FT006a	1917972	Rv1694	tlyA	33A>G	1	non-resistant associated
FT006a	2154724	Rv1908c	katG	Arg463Leu	1	non-resistant associated
FT006a	2726105	Rv2428	ahpC	-88G>A	0.979	non-resistant associated
FT006a	4242075	Rv3793	embC	Arg738Gln	0.989	non-resistant associated
FT006a	4242643	Rv3793	embC	2781C>T	1	non-resistant associated
FT006a	4407588	Rv3919c	gid	615T>C	1	non-resistant associated
FT007a	781687	Rv0682	rpsL	Lys43Arg	1	streptomycin
FT007a	7362	Rv0006	gyrA	Glu21Gln	1	non-resistant associated
FT007a	7585	Rv0006	gyrA	Ser95Thr	1	non-resistant associated
FT007a	9304	Rv0006	gyrA	Gly668Asp	1	non-resistant associated
FT007a	763031	Rv0667	rpoB	3225T>C	1	non-resistant associated
FT007a	781395	Rv0682	rpsL	-165T>C	1	non-resistant associated
FT007a	781520	Rv0682	rpsL	-40C>CAAACAGAACGTG	0.662	non-resistant associated
FT007a	1834177	Rv1630	rpsA	636A>C	1	non-resistant associated

Participant_ID	Chromosome_Position	Gene_locus	Gene	Mutation	Estimated_fraction	Drug_resistant_to
FT007a	1917972	Rv1694	tlyA	33A>G	1	non-resistant associated
FT007a	2154724	Rv1908c	katG	Arg463Leu	1	non-resistant associated
FT007a	4242643	Rv3793	embC	2781C>T	1	non-resistant associated
FT007a	4243460	Rv3794	embA	228C>T	0.978	non-resistant associated
FT007a	4326676	Rv3854c	ethA	Ser266Arg	1	non-resistant associated
FT007a	4407588	Rv3919c	gid	615T>C	1	non-resistant associated
FT007a	4407927	Rv3919c	gid	Glu92Asp	1	non-resistant associated
FT007b	781687	Rv0682	rpsL	Lys43Arg	1	streptomycin
FT007b	7362	Rv0006	gyrA	Glu21Gln	1	non-resistant associated
FT007b	7585	Rv0006	gyrA	Ser95Thr	1	non-resistant associated
FT007b	9304	Rv0006	gyrA	Gly668Asp	1	non-resistant associated
FT007b	763031	Rv0667	rpoB	3225T>C	1	non-resistant associated
FT007b	781395	Rv0682	rpsL	-165T>C	1	non-resistant associated
FT007b	781520	Rv0682	rpsL	-40C>CAAACAGAACGTG	0.709	non-resistant associated
FT007b	1834177	Rv1630	rpsA	636A>C	1	non-resistant associated
FT007b	1917972	Rv1694	tlyA	33A>G	1	non-resistant associated
FT007b	2154724	Rv1908c	katG	Arg463Leu	1	non-resistant associated
FT007b	4242643	Rv3793	embC	2781C>T	1	non-resistant associated
FT007b	4243460	Rv3794	embA	228C>T	1	non-resistant associated
FT007b	4326676	Rv3854c	ethA	Ser266Arg	0.988	non-resistant associated
FT007b	4407588	Rv3919c	gid	615T>C	1	non-resistant associated
FT007b	4407927	Rv3919c	gid	Glu92Asp	1	non-resistant associated
FT008a	6446	Rv0005	gyrB	Ala403Ser	1	non-resistant associated
FT008a	7362	Rv0006	gyrA	Glu21Gln	1	non-resistant associated
FT008a	7585	Rv0006	gyrA	Ser95Thr	1	non-resistant associated
FT008a	9143	Rv0006	gyrA	1842T>C	0.985	non-resistant associated

Participant_ID	Chromosome_Position	Gene_locus	Gene	Mutation	Estimated_fraction	Drug_resistant_to
FT008a	9304	Rv0006	gyrA	Gly668Asp	1	non-resistant associated
FT008a	9566	Rv0006	gyrA	2265C>T	0.985	non-resistant associated
FT008a	763031	Rv0667	rpoB	3225T>C	1	non-resistant associated
FT008a	763280	Rv0667	rpoB	3474C>T	1	non-resistant associated
FT008a	781395	Rv0682	rpsL	-165T>C	1	non-resistant associated
FT008a	1417554	Rv1267c	embR	-207C>G	0.989	non-resistant associated
FT008a	1673338	Rv1483	fabG1	-102G>A	0.97	non-resistant associated
FT008a	1917972	Rv1694	tlyA	33A>G	1	non-resistant associated
FT008a	2154724	Rv1908c	katG	Arg463Leu	0.987	non-resistant associated
FT008a	2518132	Rv2245	kasA	18C>T	0.986	non-resistant associated
FT008a	4240671	Rv3793	embC	Thr270Ile	1	non-resistant associated
FT008a	4241138	Rv3793	embC	Ala426Pro	0.967	non-resistant associated
FT008a	4242643	Rv3793	embC	2781C>T	1	non-resistant associated
FT008a	4244220	Rv3794	embA	988C>T	0.987	non-resistant associated
FT008a	4244635	Rv3794	embA	Val468Ala	1	non-resistant associated
FT008a	4245147	Rv3794	embA	Pro639Ser	1	non-resistant associated
FT008a	4247646	Rv3795	embB	Glu378Ala	1	non-resistant associated
FT008a	4326928	Rv3854c	ethA	546G>A	1	non-resistant associated
FT008a	4327103	Rv3854c	ethA	Gly124Asp	0.986	non-resistant associated
FT008a	4407588	Rv3919c	gid	615T>C	1	non-resistant associated
FT008b	7362	Rv0006	gyrA	Glu21Gln	1	non-resistant associated
FT008b	781395	Rv0682	rpsL	-165T>C	1	non-resistant associated
FT008b	1472150	rrs	rrs	r.305t>a	0.133	non-resistant associated
FT008b	1472172	rrs	rrs	r.327t>c	0.147	non-resistant associated
FT008b	1472203	rrs	rrs	r.358g>a	0.12	non-resistant associated
FT008b	1472598	rrs	rrs	r.753a>c	0.116	non-resistant associated
FT008b	1472616	rrs	rrs	r.771g>a	0.116	non-resistant associated
FT008b	1474001	rrl	rrl	r.344c>t	0.989	non-resistant associated
FT008b	1917972	Rv1694	tlyA	33A>G	1	non-resistant associated

**Participant_ID**	**Chromosome_Position**	**Gene_locus**	**Gene**	**Mutation**	**Estimated_fraction**	**Drug_resistant_to**
FT008b	4242643	Rv3793	embC	2781C>T	1	non-resistant associated
FT012a	7362	Rv0006	gyrA	Glu21Gln	1	non-resistant associated
FT012a	7585	Rv0006	gyrA	Ser95Thr	1	non-resistant associated
FT012a	9304	Rv0006	gyrA	Gly668Asp	1	non-resistant associated
FT012a	760115	Rv0667	rpoB	309C>T	0.991	non-resistant associated
FT012a	765150	Rv0668	rpoC	Gly594Glu	0.986	non-resistant associated
FT012a	781395	Rv0682	rpsL	-165T>C	1	non-resistant associated
FT012a	1917972	Rv1694	tlyA	33A>G	1	non-resistant associated
FT012a	4242643	Rv3793	embC	2781C>T	1	non-resistant associated
FT012a	4242803	Rv3793	embC	Val981Leu	0.983	non-resistant associated
FT012b	7362	Rv0006	gyrA	Glu21Gln	1	non-resistant associated
FT012b	7585	Rv0006	gyrA	Ser95Thr	1	non-resistant associated
FT012b	9304	Rv0006	gyrA	Gly668Asp	1	non-resistant associated
FT012b	760115	Rv0667	rpoB	309C>T	0.989	non-resistant associated
FT012b	765150	Rv0668	rpoC	Gly594Glu	0.985	non-resistant associated
FT012b	781395	Rv0682	rpsL	-165T>C	1	non-resistant associated
FT012b	1917972	Rv1694	tlyA	33A>G	1	non-resistant associated
FT012b	4242643	Rv3793	embC	2781C>T	1	non-resistant associated
FT012b	4242803	Rv3793	embC	Val981Leu	0.985	non-resistant associated
FT012c	7362	Rv0006	gyrA	Glu21Gln	1	non-resistant associated
FT012c	7585	Rv0006	gyrA	Ser95Thr	1	non-resistant associated
FT012c	9304	Rv0006	gyrA	Gly668Asp	1	non-resistant associated
FT012c	760115	Rv0667	rpoB	309C>T	1	non-resistant associated
FT012c	765150	Rv0668	rpoC	Gly594Glu	1	non-resistant associated
FT012c	781395	Rv0682	rpsL	-165T>C	1	non-resistant associated
FT012c	1917972	Rv1694	tlyA	33A>G	1	non-resistant associated
FT012c	4242643	Rv3793	embC	2781C>T	1	non-resistant associated
FT012c	4242803	Rv3793	embC	Val981Leu	0.987	non-resistant associated
FT013a	7362	Rv0006	gyrA	Glu21Gln	1	non-resistant associated

**Participant_ID**	**Chromosome_Position**	**Gene_locus**	**Gene**	**Mutation**	**Estimated_fraction**	**Drug_resistant_to**
FT013a	7585	Rv0006	gyrA	Ser95Thr	1	non-resistant associated
FT013a	9304	Rv0006	gyrA	Gly668Asp	1	non-resistant associated
FT013a	781395	Rv0682	rpsL	-165T>C	1	non-resistant associated
FT013a	1917972	Rv1694	tlyA	33A>G	1	non-resistant associated
FT013a	2289037	Rv2043c	pncA	Pro69Ser	0.97	non-resistant associated
FT013a	4242550	Rv3793	embC	2688C>G	1	non-resistant associated
FT013a	4242643	Rv3793	embC	2781C>T	1	non-resistant associated
FT013a	4326739	Rv3854c	ethA	735G>C	0.974	non-resistant associated
FT013a	4328004	Rv3855	ethR	456G>A	1	non-resistant associated
FT013b	7362	Rv0006	gyrA	Glu21Gln	1	non-resistant associated
FT013b	7585	Rv0006	gyrA	Ser95Thr	1	non-resistant associated
FT013b	9304	Rv0006	gyrA	Gly668Asp	1	non-resistant associated
FT013b	781395	Rv0682	rpsL	-165T>C	1	non-resistant associated
FT013b	1917972	Rv1694	tlyA	33A>G	1	non-resistant associated
FT013b	2289037	Rv2043c	pncA	Pro69Ser	0.988	non-resistant associated
FT013b	4242550	Rv3793	embC	2688C>G	0.984	non-resistant associated
FT013b	4242643	Rv3793	embC	2781C>T	1	non-resistant associated
FT013b	4326739	Rv3854c	ethA	735G>C	0.988	non-resistant associated
FT013b	4328004	Rv3855	ethR	456G>A	1	non-resistant associated
FT014a	781687	Rv0682	rpsL	Lys43Arg	0.961	streptomycin
FT014a	7362	Rv0006	gyrA	Glu21Gln	1	non-resistant associated
FT014a	7585	Rv0006	gyrA	Ser95Thr	0.988	non-resistant associated
FT014a	9304	Rv0006	gyrA	Gly668Asp	1	non-resistant associated
FT014a	763031	Rv0667	rpoB	3225T>C	1	non-resistant associated
FT014a	781395	Rv0682	rpsL	-165T>C	1	non-resistant associated
FT014a	781520	Rv0682	rpsL	-40C>CAAACAGAACGTG	0.699	non-resistant associated

Participant_ID	Chromosome_Position	Gene_locus	Gene	Mutation	Estimated_fraction	Drug_resistant_to
FT014a	1834177	Rv1630	rpsA	636A>C	0.99	non-resistant associated
FT014a	1917972	Rv1694	tlyA	33A>G	1	non-resistant associated
FT014a	2154724	Rv1908c	katG	Arg463Leu	0.99	non-resistant associated
FT014a	4242643	Rv3793	embC	2781C>T	1	non-resistant associated
FT014a	4243460	Rv3794	embA	228C>T	1	non-resistant associated
FT014a	4326676	Rv3854c	ethA	Ser266Arg	1	non-resistant associated
FT014a	4407588	Rv3919c	gid	615T>C	1	non-resistant associated
FT014a	4407927	Rv3919c	gid	Glu92Asp	0.99	non-resistant associated
FT014b	7362	Rv0006	gyrA	Glu21Gln	1	non-resistant associated
FT014b	7585	Rv0006	gyrA	Ser95Thr	1	non-resistant associated
FT014b	9304	Rv0006	gyrA	Gly668Asp	0.989	non-resistant associated
FT014b	763031	Rv0667	rpoB	3225T>C	0.973	non-resistant associated
FT014b	781395	Rv0682	rpsL	-165T>C	1	non-resistant associated
FT014b	1834177	Rv1630	rpsA	636A>C	0.99	non-resistant associated
FT014b	1917972	Rv1694	tlyA	33A>G	1	non-resistant associated
FT014b	2154724	Rv1908c	katG	Arg463Leu	0.991	non-resistant associated
FT014b	4242643	Rv3793	embC	2781C>T	1	non-resistant associated
FT014b	4243460	Rv3794	embA	228C>T	0.965	non-resistant associated
FT014b	4326676	Rv3854c	ethA	Ser266Arg	0.978	non-resistant associated
FT014b	4326977	Rv3854c	ethA	His166Arg	0.989	non-resistant associated
FT014b	4407588	Rv3919c	gid	615T>C	1	non-resistant associated
FT014b	4407927	Rv3919c	gid	Glu92Asp	0.989	non-resistant associated
FT015a	7362	Rv0006	gyrA	Glu21Gln	1	non-resistant associated
FT015a	7585	Rv0006	gyrA	Ser95Thr	1	non-resistant associated
FT015a	9304	Rv0006	gyrA	Gly668Asp	1	non-resistant associated
FT015a	781395	Rv0682	rpsL	-165T>C	1	non-resistant associated
FT015a	1917972	Rv1694	tlyA	33A>G	1	non-resistant associated
FT015a	2289037	Rv2043c	pncA	Pro69Ser	1	non-resistant associated
FT015a	4242550	Rv3793	embC	2688C>G	1	non-resistant associated

Participant_ID	Chromosome_Position	Gene_locus	Gene	Mutation	Estimated_fraction	Drug_resistant_to
FT015a	4242643	Rv3793	embC	2781C>T	1	non-resistant associated
FT015a	4326739	Rv3854c	ethA	735G>C	0.986	non-resistant associated
FT015a	4328004	Rv3855	ethR	456G>A	0.986	non-resistant associated
FT015b	7362	Rv0006	gyrA	Glu21Gln	1	non-resistant associated
FT015b	7585	Rv0006	gyrA	Ser95Thr	1	non-resistant associated
FT015b	9304	Rv0006	gyrA	Gly668Asp	1	non-resistant associated
FT015b	781395	Rv0682	rpsL	-165T>C	1	non-resistant associated
FT015b	1917972	Rv1694	tlyA	33A>G	1	non-resistant associated
FT015b	2289037	Rv2043c	pncA	Pro69Ser	1	non-resistant associated
FT015b	4242550	Rv3793	embC	2688C>G	1	non-resistant associated
FT015b	4242643	Rv3793	embC	2781C>T	1	non-resistant associated
FT015b	4326739	Rv3854c	ethA	735G>C	0.985	non-resistant associated
FT015b	4328004	Rv3855	ethR	456G>A	0.968	non-resistant associated
FT016a	1918647	Rv1694	tlyA	Asn236Lys	1	capreomycin
FT016a	4247451	Rv3795	embB	Ala313Val	1	ethambutol
FT016a	7362	Rv0006	gyrA	Glu21Gln	1	non-resistant associated
FT016a	7585	Rv0006	gyrA	Ser95Thr	1	non-resistant associated
FT016a	9304	Rv0006	gyrA	Gly668Asp	1	non-resistant associated
FT016a	759604	Rv0667	rpoB	-203A>G	0.98	non-resistant associated
FT016a	781395	Rv0682	rpsL	-165T>C	1	non-resistant associated
FT016a	1917972	Rv1694	tlyA	33A>G	1	non-resistant associated
FT016a	2746995	Rv2447c	folC	Val202Ile	0.977	non-resistant associated
FT016a	4242643	Rv3793	embC	2781C>T	1	non-resistant associated
FT016b	1918647	Rv1694	tlyA	Asn236Lys	0.969	capreomycin
FT016b	4247451	Rv3795	embB	Ala313Val	1	ethambutol
FT016b	7362	Rv0006	gyrA	Glu21Gln	1	non-resistant associated
FT016b	7585	Rv0006	gyrA	Ser95Thr	1	non-resistant associated
FT016b	9304	Rv0006	gyrA	Gly668Asp	1	non-resistant associated
FT016b	759604	Rv0667	rpoB	-203A>G	0.966	non-resistant associated

Participant_ID	Chromosome_Position	Gene_locus	Gene	Mutation	Estimated_fraction	Drug_resistant_to
FT016b	781395	Rv0682	rpsL	-165T>C	1	non-resistant associated
FT016b	1917972	Rv1694	tlyA	33A>G	1	non-resistant associated
FT016b	2746995	Rv2447c	folC	Val202Ile	1	non-resistant associated
FT016b	4242643	Rv3793	embC	2781C>T	1	non-resistant associated
RL001a	7362	Rv0006	gyrA	Glu21Gln	1	non-resistant associated
RL001a	7585	Rv0006	gyrA	Ser95Thr	1	non-resistant associated
RL001a	9304	Rv0006	gyrA	Gly668Asp	0.989	non-resistant associated
RL001a	781395	Rv0682	rpsL	-165T>C	1	non-resistant associated
RL001a	1834953	Rv1630	rpsA	Ala471Gly	0.974	non-resistant associated
RL001a	1917972	Rv1694	tlyA	33A>G	1	non-resistant associated
RL001a	4242643	Rv3793	embC	2781C>T	1	non-resistant associated
RL001a	4243230	Rv3794	embA	-3C>T	1	non-resistant associated
RL001a	4326739	Rv3854c	ethA	735G>C	1	non-resistant associated
RL001a	4328004	Rv3855	ethR	456G>A	1	non-resistant associated
RL001b	7362	Rv0006	gyrA	Glu21Gln	1	non-resistant associated
RL001b	7585	Rv0006	gyrA	Ser95Thr	1	non-resistant associated
RL001b	9304	Rv0006	gyrA	Gly668Asp	1	non-resistant associated
RL001b	781395	Rv0682	rpsL	-165T>C	1	non-resistant associated
RL001b	1834953	Rv1630	rpsA	Ala471Gly	0.986	non-resistant associated
RL001b	1917972	Rv1694	tlyA	33A>G	1	non-resistant associated
RL001b	4242643	Rv3793	embC	2781C>T	1	non-resistant associated
RL001b	4243230	Rv3794	embA	-3C>T	1	non-resistant associated
RL001b	4326739	Rv3854c	ethA	735G>C	1	non-resistant associated
RL001b	4328004	Rv3855	ethR	456G>A	0.982	non-resistant associated
RL002a	7362	Rv0006	gyrA	Glu21Gln	1	non-resistant associated
RL002a	7585	Rv0006	gyrA	Ser95Thr	1	non-resistant associated
RL002a	8266	Rv0006	gyrA	Ala322Glu	0.99	non-resistant associated
RL002a	9304	Rv0006	gyrA	Gly668Asp	1	non-resistant associated
RL002a	781395	Rv0682	rpsL	-165T>C	1	non-resistant associated

Participant_ID	Chromosome_Position	Gene_locus	Gene	Mutation	Estimated_fraction	Drug_resistant_to
RL002a	1917972	Rv1694	tlyA	33A>G	1	non-resistant associated
RL002a	4242643	Rv3793	embC	2781C>T	1	non-resistant associated
RL002a	7362	Rv0006	gyrA	Glu21Gln	1	non-resistant associated
RL002b	7585	Rv0006	gyrA	Ser95Thr	1	non-resistant associated
RL002b	8266	Rv0006	gyrA	Ala322Glu	0.988	non-resistant associated
RL002b	9304	Rv0006	gyrA	Gly668Asp	1	non-resistant associated
RL002b	781395	Rv0682	rpsL	-165T>C	1	non-resistant associated
RL002b	1917972	Rv1694	tlyA	33A>G	1	non-resistant associated
RL002b	4242643	Rv3793	embC	2781C>T	1	non-resistant associated
RL003a	6446	Rv0005	gyrB	Ala403Ser	1	non-resistant associated
RL003a	7362	Rv0006	gyrA	Glu21Gln	1	non-resistant associated
RL003a	7585	Rv0006	gyrA	Ser95Thr	1	non-resistant associated
RL003a	8493	Rv0006	gyrA	Leu398Phe	1	non-resistant associated
RL003a	9143	Rv0006	gyrA	1842T>C	1	non-resistant associated
RL003a	9304	Rv0006	gyrA	Gly668Asp	1	non-resistant associated
RL003a	760855	Rv0667	rpoB	Thr350Ile	1	non-resistant associated
RL003a	760969	Rv0667	rpoB	Ser388Leu	1	non-resistant associated
RL003a	761723	Rv0667	rpoB	Glu639Asp	1	non-resistant associated
RL003a	763031	Rv0667	rpoB	3225T>C	0.988	non-resistant associated
RL003a	766231	Rv0668	rpoC	2862T>C	1	non-resistant associated
RL003a	781395	Rv0682	rpsL	-165T>C	1	non-resistant associated
RL003a	1416633	Rv1267c	embR	Leu239Val	1	non-resistant associated
RL003a	1417554	Rv1267c	embR	-207C>G	1	non-resistant associated
RL003a	1674434	Rv1484	inhA	Val78Ala	1	non-resistant associated
RL003a	1917972	Rv1694	tlyA	33A>G	1	non-resistant associated
RL003a	2154724	Rv1908c	katG	Arg463Leu	1	non-resistant associated
RL003a	2155503	Rv1908c	katG	609G>A	1	non-resistant associated
RL003a	2518132	Rv2245	kasA	18C>T	1	non-resistant associated
RL003a	4240671	Rv3793	embC	Thr270Ile	1	non-resistant associated

Participant_ID	Chromosome_Position	Gene_locus	Gene	Mutation	Estimated_fraction	Drug_resistant_to
RL003a	4241843	Rv3793	embC	Leu661Ile	1	non-resistant associated
RL003a	4242643	Rv3793	embC	2781C>T	1	non-resistant associated
RL003a	4243570	Rv3794	embA	Thr113Arg	1	non-resistant associated
RL003a	4244220	Rv3794	embA	988C>T	1	non-resistant associated
RL003a	4244379	Rv3794	embA	Pro383Ser	1	non-resistant associated
RL003a	4246864	Rv3795	embB	351C>T	1	non-resistant associated
RL003a	4247646	Rv3795	embB	Glu378Ala	1	non-resistant associated
RL003a	4326465	Rv3854c	ethA	Ile337Val	1	non-resistant associated
RL003a	4327472	Rv3854c	ethA	Met1Thr	1	non-resistant associated
RL003a	4407588	Rv3919c	gid	615T>C	1	non-resistant associated
RL003a	4407966	Rv3919c	gid	Leu79Phe	1	non-resistant associated
RL003a	4408034	Rv3919c	gid	Glu57Lys	1	non-resistant associated
RL003b	7362	Rv0006	gyrA	Glu21Gln	1	non-resistant associated
RL003b	7585	Rv0006	gyrA	Ser95Thr	1	non-resistant associated
RL003b	9304	Rv0006	gyrA	Gly668Asp	1	non-resistant associated
RL003b	781395	Rv0682	rpsL	-165T>C	1	non-resistant associated
RL003b	1673393	Rv1483	fabG1	-47G>C	1	non-resistant associated
RL003b	1917972	Rv1694	tlyA	33A>G	0.979	non-resistant associated
RL003b	4242643	Rv3793	embC	2781C>T	1	non-resistant associated
RL003b	4326739	Rv3854c	ethA	735G>C	1	non-resistant associated
RL003b	4328004	Rv3855	ethR	456G>A	0.957	non-resistant associated
RL004b	7362	Rv0006	gyrA	Glu21Gln	1	non-resistant associated
RL004b	7585	Rv0006	gyrA	Ser95Thr	1	non-resistant associated
RL004b	9304	Rv0006	gyrA	Gly668Asp	0.983	non-resistant associated
RL004b	781395	Rv0682	rpsL	-165T>C	1	non-resistant associated
RL004b	1917972	Rv1694	tlyA	33A>G	1	non-resistant associated
RL004b	2289037	Rv2043c	pncA	Pro69Ser	1	non-resistant associated
RL004b	4242550	Rv3793	embC	2688C>G	1	non-resistant associated
RL004b	4242643	Rv3793	embC	2781C>T	1	non-resistant associated

Participant_ID	Chromosome_Position	Gene_locus	Gene	Mutation	Estimated_fraction	Drug_resistant_to
RL004b	4326739	Rv3854c	ethA	735G>C	1	non-resistant associated
RL004b	4328004	Rv3855	ethR	456G>A	1	non-resistant associated
RL005a	6446	Rv0005	gyrB	Ala403Ser	1	non-resistant associated
RL005a	7362	Rv0006	gyrA	Glu21Gln	1	non-resistant associated
RL005a	7585	Rv0006	gyrA	Ser95Thr	1	non-resistant associated
RL005a	9143	Rv0006	gyrA	1842T>C	1	non-resistant associated
RL005a	9304	Rv0006	gyrA	Gly668Asp	1	non-resistant associated
RL005a	9566	Rv0006	gyrA	2265C>T	1	non-resistant associated
RL005a	763031	Rv0667	rpoB	3225T>C	1	non-resistant associated
RL005a	764335	Rv0668	rpoC	966G>A	1	non-resistant associated
RL005a	781395	Rv0682	rpsL	-165T>C	1	non-resistant associated
RL005a	781676	Rv0682	rpsL	117C>T	1	non-resistant associated
RL005a	1417554	Rv1267c	embR	-207C>G	1	non-resistant associated
RL005a	1472793	rrs	rrs	r.948a>t	0.158	non-resistant associated
RL005a	1472803	rrs	rrs	r.958t>a	0.2	non-resistant associated
RL005a	1673338	Rv1483	fabG1	-102G>A	1	non-resistant associated
RL005a	1917972	Rv1694	tlyA	33A>G	1	non-resistant associated
RL005a	2154724	Rv1908c	katG	Arg463Leu	1	non-resistant associated
RL005a	2518132	Rv2245	kasA	18C>T	1	non-resistant associated
RL005a	4240671	Rv3793	embC	Thr270Ile	1	non-resistant associated
RL005a	4242643	Rv3793	embC	2781C>T	1	non-resistant associated
RL005a	4244220	Rv3794	embA	988C>T	1	non-resistant associated
RL005a	4244635	Rv3794	embA	Val468Ala	1	non-resistant associated
RL005a	4245147	Rv3794	embA	Pro639Ser	1	non-resistant associated
RL005a	4247646	Rv3795	embB	Glu378Ala	1	non-resistant associated
RL005a	4326928	Rv3854c	ethA	546G>A	1	non-resistant associated
RL005a	4327103	Rv3854c	ethA	Gly124Asp	1	non-resistant associated
RL005a	4407588	Rv3919c	gid	615T>C	1	non-resistant associated
RL005b	781687	Rv0682	rpsL	Lys43Arg	1	streptomycin

Participant_ID	Chromosome_Position	Gene_locus	Gene	Mutation	Estimated_fraction	Drug_resistant_to
RL005b	6446	Rv0005	gyrB	Ala403Ser	1	non-resistant associated
RL005b	7362	Rv0006	gyrA	Glu21Gln	1	non-resistant associated
RL005b	7585	Rv0006	gyrA	Ser95Thr	1	non-resistant associated
RL005b	9143	Rv0006	gyrA	1842T>C	1	non-resistant associated
RL005b	9304	Rv0006	gyrA	Gly668Asp	1	non-resistant associated
RL005b	9566	Rv0006	gyrA	2265C>T	1	non-resistant associated
RL005b	763031	Rv0667	rpoB	3225T>C	1	non-resistant associated
RL005b	781395	Rv0682	rpsL	-165T>C	1	non-resistant associated
RL005b	800930	Rv0701	rplC	Thr41Met	1	non-resistant associated
RL005b	1417554	Rv1267c	embR	-207C>G	1	non-resistant associated
RL005b	1673338	Rv1483	fabG1	-102G>A	1	non-resistant associated
RL005b	1917972	Rv1694	tlyA	33A>G	1	non-resistant associated
RL005b	2154724	Rv1908c	katG	Arg463Leu	1	non-resistant associated
RL005b	2518132	Rv2245	kasA	18C>T	1	non-resistant associated
RL005b	4240671	Rv3793	embC	Thr270Ile	1	non-resistant associated
RL005b	4242643	Rv3793	embC	2781C>T	1	non-resistant associated
RL005b	4244220	Rv3794	embA	988C>T	1	non-resistant associated
RL005b	4244635	Rv3794	embA	Val468Ala	1	non-resistant associated
RL005b	4245147	Rv3794	embA	Pro639Ser	1	non-resistant associated
RL005b	4247646	Rv3795	embB	Glu378Ala	1	non-resistant associated
RL005b	4326928	Rv3854c	ethA	546G>A	1	non-resistant associated
RL005b	4327103	Rv3854c	ethA	Gly124Asp	1	non-resistant associated
RL005b	4407588	Rv3919c	gid	615T>C	1	non-resistant associated
RL006a	1918647	Rv1694	tlyA	Asn236Lys	0.988	capreomycin
RL006a	4247451	Rv3795	embB	Ala313Val	0.989	ethambutol
RL006a	7362	Rv0006	gyrA	Glu21Gln	0.989	non-resistant associated
RL006a	7585	Rv0006	gyrA	Ser95Thr	0.989	non-resistant associated
RL006a	9304	Rv0006	gyrA	Gly668Asp	1	non-resistant associated
RL006a	759604	Rv0667	rpoB	-203A>G	1	non-resistant associated

Participant_ID	Chromosome_Position	Gene_locus	Gene	Mutation	Estimated_fraction	Drug_resistant_to
RL006a	781395	Rv0682	rpsL	-165T>C	1	non-resistant associated
RL006a	1917972	Rv1694	tlyA	33A>G	1	non-resistant associated
RL006a	2746995	Rv2447c	folC	Val202Ile	0.988	non-resistant associated
RL006a	4242643	Rv3793	embC	2781C>T	1	non-resistant associated
RL007a	6446	Rv0005	gyrB	Ala403Ser	1	non-resistant associated
RL007a	7362	Rv0006	gyrA	Glu21Gln	1	non-resistant associated
RL007a	7585	Rv0006	gyrA	Ser95Thr	1	non-resistant associated
RL007a	9143	Rv0006	gyrA	1842T>C	1	non-resistant associated
RL007a	9304	Rv0006	gyrA	Gly668Asp	1	non-resistant associated
RL007a	9566	Rv0006	gyrA	2265C>T	1	non-resistant associated
RL007a	763031	Rv0667	rpoB	3225T>C	1	non-resistant associated
RL007a	763280	Rv0667	rpoB	3474C>T	1	non-resistant associated
RL007a	781395	Rv0682	rpsL	-165T>C	1	non-resistant associated
RL007a	1417554	Rv1267c	embR	-207C>G	1	non-resistant associated
RL007a	1476383	rrl	rrl	r.2726t>a	0.2	non-resistant associated
RL007a	1476408	rrl	rrl	r.2751g>a	0.2	non-resistant associated
RL007a	1476466	rrl	rrl	r.2809c>t	0.182	non-resistant associated
RL007a	1476481	rrl	rrl	r.2824t>c	0.167	non-resistant associated
RL007a	1673338	Rv1483	fabG1	-102G>A	1	non-resistant associated
RL007a	1917972	Rv1694	tlyA	33A>G	1	non-resistant associated
RL007a	2154724	Rv1908c	katG	Arg463Leu	1	non-resistant associated
RL007a	2518132	Rv2245	kasA	18C>T	1	non-resistant associated
RL007a	2715154	Rv2416c	eis	Gly60Glu	1	non-resistant associated
RL007a	4240671	Rv3793	embC	Thr270Ile	1	non-resistant associated
RL007a	4242643	Rv3793	embC	2781C>T	1	non-resistant associated
RL007a	4244220	Rv3794	embA	988C>T	1	non-resistant associated
RL007a	4244635	Rv3794	embA	Val468Ala	0.993	non-resistant associated
RL007a	4245147	Rv3794	embA	Pro639Ser	1	non-resistant associated
RL007a	4247646	Rv3795	embB	Glu378Ala	1	non-resistant associated

Participant_ID	Chromosome_Position	Gene_locus	Gene	Mutation	Estimated_fraction	Drug_resistant_to
RL007a	4326928	Rv3854c	ethA	546G>A	1	non-resistant associated
RL007a	4327103	Rv3854c	ethA	Gly124Asp	1	non-resistant associated
RL007a	4407588	Rv3919c	gid	615T>C	0.992	non-resistant associated
RL007b	6446	Rv0005	gyrB	Ala403Ser	1	non-resistant associated
RL007b	7362	Rv0006	gyrA	Glu21Gln	1	non-resistant associated
RL007b	7585	Rv0006	gyrA	Ser95Thr	1	non-resistant associated
RL007b	9143	Rv0006	gyrA	1842T>C	0.986	non-resistant associated
RL007b	9304	Rv0006	gyrA	Gly668Asp	1	non-resistant associated
RL007b	9566	Rv0006	gyrA	2265C>T	0.974	non-resistant associated
RL007b	763031	Rv0667	rpoB	3225T>C	1	non-resistant associated
RL007b	763280	Rv0667	rpoB	3474C>T	1	non-resistant associated
RL007b	781395	Rv0682	rpsL	-165T>C	1	non-resistant associated
RL007b	1417554	Rv1267c	embR	-207C>G	0.987	non-resistant associated
RL007b	1673338	Rv1483	fabG1	-102G>A	0.987	non-resistant associated
RL007b	1917972	Rv1694	tlyA	33A>G	1	non-resistant associated
RL007b	2154724	Rv1908c	katG	Arg463Leu	1	non-resistant associated
RL007b	2518132	Rv2245	kasA	18C>T	1	non-resistant associated
RL007b	4240671	Rv3793	embC	Thr270Ile	0.983	non-resistant associated
RL007b	4242643	Rv3793	embC	2781C>T	1	non-resistant associated
RL007b	4244220	Rv3794	embA	988C>T	1	non-resistant associated
RL007b	4244635	Rv3794	embA	Val468Ala	0.971	non-resistant associated
RL007b	4245147	Rv3794	embA	Pro639Ser	0.981	non-resistant associated
RL007b	4247646	Rv3795	embB	Glu378Ala	1	non-resistant associated
RL007b	4326928	Rv3854c	ethA	546G>A	1	non-resistant associated
RL007b	4327103	Rv3854c	ethA	Gly124Asp	1	non-resistant associated
RL007b	4407588	Rv3919c	gid	615T>C	1	non-resistant associated
RL008a	6467	Rv0005	gyrB	Ala410Thr	0.986	non-resistant associated
RL008a	7362	Rv0006	gyrA	Glu21Gln	1	non-resistant associated
RL008a	781395	Rv0682	rpsL	-165T>C	1	non-resistant associated

Participant_ID	Chromosome_Position	Gene_locus	Gene	Mutation	Estimated_fraction	Drug_resistant_to
RL008a	1474001	rrl	rrl	r.344c>t	0.965	non-resistant associated
RL008a	1917972	Rv1694	tlyA	33A>G	1	non-resistant associated
RL008a	4242643	Rv3793	embC	2781C>T	1	non-resistant associated
RL008b	7362	Rv0006	gyrA	Glu21Gln	1	non-resistant associated
RL008b	7585	Rv0006	gyrA	Ser95Thr	1	non-resistant associated
RL008b	9304	Rv0006	gyrA	Gly668Asp	1	non-resistant associated
RL008b	781395	Rv0682	rpsL	-165T>C	1	non-resistant associated
RL008b	1917972	Rv1694	tlyA	33A>G	1	non-resistant associated
RL008b	4242643	Rv3793	embC	2781C>T	1	non-resistant associated
RL008b	4248767	Rv3795	embB	Asp752His	1	non-resistant associated
RL008b	4326739	Rv3854c	ethA	735G>C	1	non-resistant associated
RL008b	4328004	Rv3855	ethR	456G>A	1	non-resistant associated
RL009a	7362	Rv0006	gyrA	Glu21Gln	1	non-resistant associated
RL009a	7585	Rv0006	gyrA	Ser95Thr	1	non-resistant associated
RL009a	9304	Rv0006	gyrA	Gly668Asp	1	non-resistant associated
RL009a	781395	Rv0682	rpsL	-165T>C	1	non-resistant associated
RL009a	1834873	Rv1630	rpsA	1332C>A	0.99	non-resistant associated
RL009a	1917972	Rv1694	tlyA	33A>G	1	non-resistant associated
RL009a	4242550	Rv3793	embC	2688C>G	1	non-resistant associated
RL009a	4242643	Rv3793	embC	2781C>T	1	non-resistant associated
RL009a	4326739	Rv3854c	ethA	735G>C	1	non-resistant associated
RL009a	4328004	Rv3855	ethR	456G>A	1	non-resistant associated
RL009b	7362	Rv0006	gyrA	Glu21Gln	1	non-resistant associated
RL009b	7585	Rv0006	gyrA	Ser95Thr	1	non-resistant associated
RL009b	9304	Rv0006	gyrA	Gly668Asp	1	non-resistant associated
RL009b	781395	Rv0682	rpsL	-165T>C	1	non-resistant associated
RL009b	1834873	Rv1630	rpsA	1332C>A	0.986	non-resistant associated
RL009b	1917972	Rv1694	tlyA	33A>G	0.984	non-resistant associated
RL009b	4242550	Rv3793	embC	2688C>G	0.983	non-resistant associated

Participant_ID	Chromosome_Position	Gene_locus	Gene	Mutation	Estimated_fraction	Drug_resistant_to
RL009b	4242643	Rv3793	embC	2781C>T	1	non-resistant associated
RL009b	4326739	Rv3854c	ethA	735G>C	1	non-resistant associated
RL009b	4328004	Rv3855	ethR	456G>A	1	non-resistant associated
RL010a	7362	Rv0006	gyrA	Glu21Gln	1	non-resistant associated
RL010a	7585	Rv0006	gyrA	Ser95Thr	1	non-resistant associated
RL010a	9304	Rv0006	gyrA	Gly668Asp	1	non-resistant associated
RL010a	781395	Rv0682	rpsL	-165T>C	1	non-resistant associated
RL010a	1917972	Rv1694	tlyA	33A>G	1	non-resistant associated
RL010a	2289037	Rv2043c	pncA	Pro69Ser	1	non-resistant associated
RL010a	4242550	Rv3793	embC	2688C>G	1	non-resistant associated
RL010a	4242643	Rv3793	embC	2781C>T	1	non-resistant associated
RL010a	4326739	Rv3854c	ethA	735G>C	1	non-resistant associated
RL010a	4328004	Rv3855	ethR	456G>A	1	non-resistant associated
RL010b	7362	Rv0006	gyrA	Glu21Gln	1	non-resistant associated
RL010b	7585	Rv0006	gyrA	Ser95Thr	1	non-resistant associated
RL010b	9304	Rv0006	gyrA	Gly668Asp	1	non-resistant associated
RL010b	781395	Rv0682	rpsL	-165T>C	1	non-resistant associated
RL010b	1917972	Rv1694	tlyA	33A>G	1	non-resistant associated
RL010b	2289037	Rv2043c	pncA	Pro69Ser	1	non-resistant associated
RL010b	4242550	Rv3793	embC	2688C>G	1	non-resistant associated
RL010b	4242643	Rv3793	embC	2781C>T	1	non-resistant associated
RL010b	4326739	Rv3854c	ethA	735G>C	1	non-resistant associated
RL010b	4328004	Rv3855	ethR	456G>A	1	non-resistant associated
RL011a	7362	Rv0006	gyrA	Glu21Gln	1	non-resistant associated
RL011a	7585	Rv0006	gyrA	Ser95Thr	1	non-resistant associated
RL011a	9304	Rv0006	gyrA	Gly668Asp	1	non-resistant associated
RL011a	781395	Rv0682	rpsL	-165T>C	0.986	non-resistant associated
RL011a	1673393	Rv1483	fabG1	-47G>C	1	non-resistant associated
RL011a	1917972	Rv1694	tlyA	33A>G	1	non-resistant associated

Participant_ID	Chromosome_Position	Gene_locus	Gene	Mutation	Estimated_fraction	Drug_resistant_to
RL011a	4242643	Rv3793	embC	2781C>T	1	non-resistant associated
RL011a	4326739	Rv3854c	ethA	735G>C	1	non-resistant associated
RL011a	4328004	Rv3855	ethR	456G>A	1	non-resistant associated
RL011b	7362	Rv0006	gyrA	Glu21Gln	1	non-resistant associated
RL011b	7585	Rv0006	gyrA	Ser95Thr	1	non-resistant associated
RL011b	9304	Rv0006	gyrA	Gly668Asp	1	non-resistant associated
RL011b	781395	Rv0682	rpsL	-165T>C	1	non-resistant associated
RL011b	1673393	Rv1483	fabG1	-47G>C	0.954	non-resistant associated
RL011b	1917972	Rv1694	tlyA	33A>G	1	non-resistant associated
RL011b	4242643	Rv3793	embC	2781C>T	1	non-resistant associated
RL011b	4326739	Rv3854c	ethA	735G>C	1	non-resistant associated
RL011b	4328004	Rv3855	ethR	456G>A	1	non-resistant associated
RL011c	7362	Rv0006	gyrA	Glu21Gln	1	non-resistant associated
RL011c	7585	Rv0006	gyrA	Ser95Thr	1	non-resistant associated
RL011c	9304	Rv0006	gyrA	Gly668Asp	1	non-resistant associated
RL011c	781395	Rv0682	rpsL	-165T>C	1	non-resistant associated
RL011c	1673393	Rv1483	fabG1	-47G>C	1	non-resistant associated
RL011c	1917972	Rv1694	tlyA	33A>G	1	non-resistant associated
RL011c	4242643	Rv3793	embC	2781C>T	1	non-resistant associated
RL011c	4326739	Rv3854c	ethA	735G>C	1	non-resistant associated
RL011c	4328004	Rv3855	ethR	456G>A	1	non-resistant associated
RL012a	7362	Rv0006	gyrA	Glu21Gln	1	non-resistant associated
RL012a	7585	Rv0006	gyrA	Ser95Thr	1	non-resistant associated
RL012a	9304	Rv0006	gyrA	Gly668Asp	1	non-resistant associated
RL012a	763031	Rv0667	rpoB	3225T>C	1	non-resistant associated
RL012a	781395	Rv0682	rpsL	-165T>C	0.992	non-resistant associated
RL012a	1834177	Rv1630	rpsA	636A>C	0.983	non-resistant associated
RL012a	1917972	Rv1694	tlyA	33A>G	1	non-resistant associated
RL012a	2154724	Rv1908c	katG	Arg463Leu	0.991	non-resistant associated

Participant_ID	Chromosome_Position	Gene_locus	Gene	Mutation	Estimated_fraction	Drug_resistant_to
RL012a	4242643	Rv3793	embC	2781C>T	1	non-resistant associated
RL012a	4243460	Rv3794	embA	228C>T	1	non-resistant associated
RL012a	4326676	Rv3854c	ethA	Ser266Arg	0.989	non-resistant associated
RL012a	4326977	Rv3854c	ethA	His166Arg	0.99	non-resistant associated
RL012a	4407588	Rv3919c	gid	615T>C	0.989	non-resistant associated
RL012a	4407927	Rv3919c	gid	Glu92Asp	0.98	non-resistant associated
RL012b	7362	Rv0006	gyrA	Glu21Gln	1	non-resistant associated
RL012b	7585	Rv0006	gyrA	Ser95Thr	1	non-resistant associated
RL012b	9304	Rv0006	gyrA	Gly668Asp	1	non-resistant associated
RL012b	763031	Rv0667	rpoB	3225T>C	1	non-resistant associated
RL012b	781395	Rv0682	rpsL	-165T>C	1	non-resistant associated
RL012b	1834177	Rv1630	rpsA	636A>C	1	non-resistant associated
RL012b	1917972	Rv1694	tlyA	33A>G	1	non-resistant associated
RL012b	2154724	Rv1908c	katG	Arg463Leu	0.99	non-resistant associated
RL012b	4242643	Rv3793	embC	2781C>T	1	non-resistant associated
RL012b	4243460	Rv3794	embA	228C>T	0.99	non-resistant associated
RL012b	4326676	Rv3854c	ethA	Ser266Arg	1	non-resistant associated
RL012b	4326977	Rv3854c	ethA	His166Arg	1	non-resistant associated
RL012b	4407588	Rv3919c	gid	615T>C	1	non-resistant associated
RL012b	4407927	Rv3919c	gid	Glu92Asp	0.988	non-resistant associated
RL013a	7362	Rv0006	gyrA	Glu21Gln	1	non-resistant associated
RL013a	7585	Rv0006	gyrA	Ser95Thr	1	non-resistant associated
RL013a	9304	Rv0006	gyrA	Gly668Asp	1	non-resistant associated
RL013a	781395	Rv0682	rpsL	-165T>C	1	non-resistant associated
RL013a	1917972	Rv1694	tlyA	33A>G	1	non-resistant associated
RL013a	2289037	Rv2043c	pncA	Pro69Ser	1	non-resistant associated
RL013a	4242550	Rv3793	embC	2688C>G	1	non-resistant associated
RL013a	4242643	Rv3793	embC	2781C>T	1	non-resistant associated
RL013a	4326739	Rv3854c	ethA	735G>C	1	non-resistant associated

Participant_ID	Chromosome_Position	Gene_locus	Gene	Mutation	Estimated_fraction	Drug_resistant_to
RL013a	4328004	Rv3855	ethR	456G>A	0.98	non-resistant associated
RL013b	7362	Rv0006	gyrA	Glu21Gln	1	non-resistant associated
RL013b	7585	Rv0006	gyrA	Ser95Thr	1	non-resistant associated
RL013b	9304	Rv0006	gyrA	Gly668Asp	1	non-resistant associated
RL013b	781395	Rv0682	rpsL	-165T>C	1	non-resistant associated
RL013b	1917972	Rv1694	tlyA	33A>G	1	non-resistant associated
RL013b	2289037	Rv2043c	pncA	Pro69Ser	1	non-resistant associated
RL013b	4242550	Rv3793	embC	2688C>G	1	non-resistant associated
RL013b	4242643	Rv3793	embC	2781C>T	1	non-resistant associated
RL013b	4326739	Rv3854c	ethA	735G>C	1	non-resistant associated
RL013b	4328004	Rv3855	ethR	456G>A	1	non-resistant associated
RL014a	7362	Rv0006	gyrA	Glu21Gln	1	non-resistant associated
RL014a	7585	Rv0006	gyrA	Ser95Thr	1	non-resistant associated
RL014a	9304	Rv0006	gyrA	Gly668Asp	1	non-resistant associated
RL014a	781395	Rv0682	rpsL	-165T>C	1	non-resistant associated
RL014a	1917972	Rv1694	tlyA	33A>G	1	non-resistant associated
RL014a	3074465	Rv2764c	thyA	Pro3Ala	0.19	non-resistant associated
RL014a	4242643	Rv3793	embC	2781C>T	1	non-resistant associated
RL014a	4247717	Rv3795	embB	Leu402Val	0.97	non-resistant associated
RL014a	4248767	Rv3795	embB	Asp752His	1	non-resistant associated
RL014a	4326739	Rv3854c	ethA	735G>C	1	non-resistant associated
RL014a	4328004	Rv3855	ethR	456G>A	0.984	non-resistant associated
RL014b	7362	Rv0006	gyrA	Glu21Gln	1	non-resistant associated
RL014b	7585	Rv0006	gyrA	Ser95Thr	1	non-resistant associated
RL014b	9304	Rv0006	gyrA	Gly668Asp	1	non-resistant associated
RL014b	781395	Rv0682	rpsL	-165T>C	1	non-resistant associated
RL014b	1917972	Rv1694	tlyA	33A>G	1	non-resistant associated
RL014b	4242643	Rv3793	embC	2781C>T	1	non-resistant associated
RL014b	4247717	Rv3795	embB	Leu402Val	0.986	non-resistant associated

Participant_ID	Chromosome_Position	Gene_locus	Gene	Mutation	Estimated_fraction	Drug_resistant_to
RL014b	4248767	Rv3795	embB	Asp752His	1	non-resistant associated
RL014b	4326739	Rv3854c	ethA	735G>C	1	non-resistant associated
RL014b	4328004	Rv3855	ethR	456G>A	1	non-resistant associated
RL015a	7362	Rv0006	gyrA	Glu21Gln	1	non-resistant associated
RL015a	7585	Rv0006	gyrA	Ser95Thr	1	non-resistant associated
RL015a	9304	Rv0006	gyrA	Gly668Asp	1	non-resistant associated
RL015a	781395	Rv0682	rpsL	-165T>C	1	non-resistant associated
RL015a	1917972	Rv1694	tlyA	33A>G	1	non-resistant associated
RL015a	4242550	Rv3793	embC	2688C>G	0.986	non-resistant associated
RL015a	4242643	Rv3793	embC	2781C>T	1	non-resistant associated
RL015a	4326739	Rv3854c	ethA	735G>C	1	non-resistant associated
RL015a	4328004	Rv3855	ethR	456G>A	1	non-resistant associated
RL015b	4327876	Rv3855	ethR	Phe110Leu	1	ethionamide
RL015b	7362	Rv0006	gyrA	Glu21Gln	1	non-resistant associated
RL015b	7585	Rv0006	gyrA	Ser95Thr	1	non-resistant associated
RL015b	9304	Rv0006	gyrA	Gly668Asp	1	non-resistant associated
RL015b	760115	Rv0667	rpoB	309C>T	0.973	non-resistant associated
RL015b	765150	Rv0668	rpoC	Gly594Glu	1	non-resistant associated
RL015b	781395	Rv0682	rpsL	-165T>C	1	non-resistant associated
RL015b	800889	Rv0701	rplC	81C>A	1	non-resistant associated
RL015b	1472580	rrs	rrs	r.735c>g	1	non-resistant associated
RL015b	1917972	Rv1694	tlyA	33A>G	1	non-resistant associated
RL015b	4242643	Rv3793	embC	2781C>T	1	non-resistant associated
RL015b	4242803	Rv3793	embC	Val981Leu	1	non-resistant associated
RL020a	7362	Rv0006	gyrA	Glu21Gln	1	non-resistant associated
RL020a	7585	Rv0006	gyrA	Ser95Thr	1	non-resistant associated
RL020a	9304	Rv0006	gyrA	Gly668Asp	1	non-resistant associated
RL020a	760115	Rv0667	rpoB	309C>T	0.95	non-resistant associated
RL020a	765150	Rv0668	rpoC	Gly594Glu	0.958	non-resistant associated

Participant_ID	Chromosome_Position	Gene_locus	Gene	Mutation	Estimated_fraction	Drug_resistant_to
RL020a	781395	Rv0682	rpsL	-165T>C	1	non-resistant associated
RL020a	1917972	Rv1694	tlyA	33A>G	1	non-resistant associated
RL020a	4242643	Rv3793	embC	2781C>T	1	non-resistant associated
RL020a	4242803	Rv3793	embC	Val981Leu	0.98	non-resistant associated
RL020a	4245298	Rv3794	embA	Pro689Leu	1	non-resistant associated
RL020a	4408135	Rv3919c	gid	Ala23Asp	0.962	non-resistant associated
RL020b	7362	Rv0006	gyrA	Glu21Gln	1	non-resistant associated
RL020b	7585	Rv0006	gyrA	Ser95Thr	0.992	non-resistant associated
RL020b	9304	Rv0006	gyrA	Gly668Asp	1	non-resistant associated
RL020b	760115	Rv0667	rpoB	309C>T	1	non-resistant associated
RL020b	765150	Rv0668	rpoC	Gly594Glu	1	non-resistant associated
RL020b	781395	Rv0682	rpsL	-165T>C	1	non-resistant associated
RL020b	1917972	Rv1694	tlyA	33A>G	1	non-resistant associated
RL020b	4242643	Rv3793	embC	2781C>T	1	non-resistant associated
RL020b	4242803	Rv3793	embC	Val981Leu	1	non-resistant associated
RL020b	4245298	Rv3794	embA	Pro689Leu	1	non-resistant associated
RL020b	4408135	Rv3919c	gid	Ala23Asp	1	non-resistant associated
RL021a	7362	Rv0006	gyrA	Glu21Gln	1	non-resistant associated
RL021a	7585	Rv0006	gyrA	Ser95Thr	0.986	non-resistant associated
RL021a	9304	Rv0006	gyrA	Gly668Asp	1	non-resistant associated
RL021a	781395	Rv0682	rpsL	-165T>C	1	non-resistant associated
RL021a	1917972	Rv1694	tlyA	33A>G	1	non-resistant associated
RL021a	2289037	Rv2043c	pncA	Pro69Ser	1	non-resistant associated
RL021a	4242550	Rv3793	embC	2688C>G	1	non-resistant associated
RL021a	4242643	Rv3793	embC	2781C>T	1	non-resistant associated
RL021a	4326739	Rv3854c	ethA	735G>C	1	non-resistant associated
RL021a	4328004	Rv3855	ethR	456G>A	1	non-resistant associated
RL022a	7362	Rv0006	gyrA	Glu21Gln	1	non-resistant associated
RL022a	7585	Rv0006	gyrA	Ser95Thr	0.988	non-resistant associated

Participant_ID	Chromosome_Position	Gene_locus	Gene	Mutation	Estimated_fraction	Drug_resistant_to
RL022a	9304	Rv0006	gyrA	Gly668Asp	1	non-resistant associated
RL022a	781395	Rv0682	rpsL	-165T>C	1	non-resistant associated
RL022a	1917972	Rv1694	tlyA	33A>G	1	non-resistant associated
RL022a	2289037	Rv2043c	pncA	Pro69Ser	1	non-resistant associated
RL022a	4242550	Rv3793	embC	2688C>G	1	non-resistant associated
RL022a	4242643	Rv3793	embC	2781C>T	1	non-resistant associated
RL022a	4326739	Rv3854c	ethA	735G>C	1	non-resistant associated
RL022a	4328004	Rv3855	ethR	456G>A	1	non-resistant associated
RL022b	7362	Rv0006	gyrA	Glu21Gln	1	non-resistant associated
RL022b	7585	Rv0006	gyrA	Ser95Thr	1	non-resistant associated
RL022b	9304	Rv0006	gyrA	Gly668Asp	1	non-resistant associated
RL022b	781395	Rv0682	rpsL	-165T>C	1	non-resistant associated
RL022b	1917972	Rv1694	tlyA	33A>G	1	non-resistant associated
RL022b	2289037	Rv2043c	pncA	Pro69Ser	1	non-resistant associated
RL022b	4242550	Rv3793	embC	2688C>G	1	non-resistant associated
RL022b	4242643	Rv3793	embC	2781C>T	1	non-resistant associated
RL022b	4326739	Rv3854c	ethA	735G>C	1	non-resistant associated
RL022b	4328004	Rv3855	ethR	456G>A	1	non-resistant associated
RL022c	7362	Rv0006	gyrA	Glu21Gln	1	non-resistant associated
RL022c	7585	Rv0006	gyrA	Ser95Thr	1	non-resistant associated
RL022c	9304	Rv0006	gyrA	Gly668Asp	1	non-resistant associated
RL022c	781395	Rv0682	rpsL	-165T>C	1	non-resistant associated
RL022c	1917972	Rv1694	tlyA	33A>G	1	non-resistant associated
RL022c	2289037	Rv2043c	pncA	Pro69Ser	0.976	non-resistant associated
RL022c	4242550	Rv3793	embC	2688C>G	1	non-resistant associated
RL022c	4242643	Rv3793	embC	2781C>T	1	non-resistant associated
RL022c	4326739	Rv3854c	ethA	735G>C	1	non-resistant associated
RL022c	4328004	Rv3855	ethR	456G>A	1	non-resistant associated
RL023a	7362	Rv0006	gyrA	Glu21Gln	1	non-resistant associated

Participant_ID	Chromosome_Position	Gene_locus	Gene	Mutation	Estimated_fraction	Drug_resistant_to
RL023a	781395	Rv0682	rpsL	-165T>C	1	non-resistant associated
RL023a	1917972	Rv1694	tlyA	33A>G	1	non-resistant associated
RL023a	2154980	Rv1908c	katG	1132G>A	1	non-resistant associated
RL023a	4242643	Rv3793	embC	2781C>T	1	non-resistant associated
RL023b	7362	Rv0006	gyrA	Glu21Gln	1	non-resistant associated
RL023b	781395	Rv0682	rpsL	-165T>C	1	non-resistant associated
RL023b	1917972	Rv1694	tlyA	33A>G	0.983	non-resistant associated
RL023b	2154980	Rv1908c	katG	1132G>A	1	non-resistant associated
RL023b	4242643	Rv3793	embC	2781C>T	1	non-resistant associated
RL024a	7362	Rv0006	gyrA	Glu21Gln	1	non-resistant associated
RL024a	7585	Rv0006	gyrA	Ser95Thr	1	non-resistant associated
RL024a	9304	Rv0006	gyrA	Gly668Asp	1	non-resistant associated
RL024a	781395	Rv0682	rpsL	-165T>C	1	non-resistant associated
RL024a	1917972	Rv1694	tlyA	33A>G	1	non-resistant associated
RL024a	2155389	Rv1908c	katG	723G>C	1	non-resistant associated
RL024a	4242550	Rv3793	embC	2688C>G	1	non-resistant associated
RL024a	4242643	Rv3793	embC	2781C>T	1	non-resistant associated
RL024a	4326739	Rv3854c	ethA	735G>C	1	non-resistant associated
RL024a	4328004	Rv3855	ethR	456G>A	1	non-resistant associated
RL024b	7362	Rv0006	gyrA	Glu21Gln	1	non-resistant associated
RL024b	7585	Rv0006	gyrA	Ser95Thr	1	non-resistant associated
RL024b	9304	Rv0006	gyrA	Gly668Asp	1	non-resistant associated
RL024b	781395	Rv0682	rpsL	-165T>C	1	non-resistant associated
RL024b	1917972	Rv1694	tlyA	33A>G	1	non-resistant associated
RL024b	2155389	Rv1908c	katG	723G>C	1	non-resistant associated
RL024b	4242550	Rv3793	embC	2688C>G	0.983	non-resistant associated
RL024b	4242643	Rv3793	embC	2781C>T	1	non-resistant associated
RL024b	4326739	Rv3854c	ethA	735G>C	0.984	non-resistant associated
RL024b	4328004	Rv3855	ethR	456G>A	1	non-resistant associated

Participant_ID	Chromosome_Position	Gene_locus	Gene	Mutation	Estimated_fraction	Drug_resistant_to
RL025a	4247431	Rv3795	embB	Met306Ile	1	ethambutol
RL025a	2288847	Rv2043c	pncA	Gly132Ala	1	pyrazinamide
RL025a	7362	Rv0006	gyrA	Glu21Gln	1	non-resistant associated
RL025a	7585	Rv0006	gyrA	Ser95Thr	1	non-resistant associated
RL025a	9304	Rv0006	gyrA	Gly668Asp	1	non-resistant associated
RL025a	9557	Rv0006	gyrA	2256G>C	1	non-resistant associated
RL025a	765150	Rv0668	rpoC	Gly594Glu	1	non-resistant associated
RL025a	781395	Rv0682	rpsL	-165T>C	1	non-resistant associated
RL025a	781421	Rv0682	rpsL	-139C>A	0.988	non-resistant associated
RL025a	1674048	Rv1483	fabG1	609G>A	0.988	non-resistant associated
RL025a	1917972	Rv1694	tlyA	33A>G	1	non-resistant associated
RL025a	4242643	Rv3793	embC	2781C>T	1	non-resistant associated
RL025a	4242803	Rv3793	embC	Val981Leu	0.989	non-resistant associated
RL025a	4407967	Rv3919c	gid	Leu79Ser	1	non-resistant associated
RL025b	4247431	Rv3795	embB	Met306Ile	0.984	ethambutol
RL025b	2288847	Rv2043c	pncA	Gly132Ala	0.98	pyrazinamide
RL025b	7362	Rv0006	gyrA	Glu21Gln	1	non-resistant associated
RL025b	7585	Rv0006	gyrA	Ser95Thr	1	non-resistant associated
RL025b	9304	Rv0006	gyrA	Gly668Asp	0.979	non-resistant associated
RL025b	9557	Rv0006	gyrA	2256G>C	1	non-resistant associated
RL025b	765150	Rv0668	rpoC	Gly594Glu	0.983	non-resistant associated
RL025b	781395	Rv0682	rpsL	-165T>C	1	non-resistant associated
RL025b	781421	Rv0682	rpsL	-139C>A	1	non-resistant associated
RL025b	1416982	Rv1267c	embR	365 365del	1	non-resistant associated
RL025b	1674048	Rv1483	fabG1	609G>A	1	non-resistant associated
RL025b	1917972	Rv1694	tlyA	33A>G	1	non-resistant associated
RL025b	4242643	Rv3793	embC	2781C>T	1	non-resistant associated
RL025b	4242803	Rv3793	embC	Val981Leu	1	non-resistant associated
RL025b	4407967	Rv3919c	gid	Leu79Ser	1	non-resistant associated

Participant_ID	Chromosome_Position	Gene_locus	Gene	Mutation	Estimated_fraction	Drug_resistant_to
RL026a	4408100	Rv3919c	gid	102 102del	1	streptomycin
RL026a	7362	Rv0006	gyrA	Glu21Gln	1	non-resistant associated
RL026a	7585	Rv0006	gyrA	Ser95Thr	1	non-resistant associated
RL026a	9304	Rv0006	gyrA	Gly668Asp	1	non-resistant associated
RL026a	781395	Rv0682	rpsL	-165T>C	1	non-resistant associated
RL026a	1917972	Rv1694	tlyA	33A>G	1	non-resistant associated
RL026a	4242643	Rv3793	embC	2781C>T	1	non-resistant associated
RL026a	4326739	Rv3854c	ethA	735G>C	1	non-resistant associated
RL026a	4328004	Rv3855	ethR	456G>A	1	non-resistant associated
RL026a	4408101	Rv3919c	gid	r.102c>g	0.3	non-resistant associated
RL026b	7362	Rv0006	gyrA	Glu21Gln	1	non-resistant associated
RL026b	7585	Rv0006	gyrA	Ser95Thr	1	non-resistant associated
RL026b	9304	Rv0006	gyrA	Gly668Asp	1	non-resistant associated
RL026b	781395	Rv0682	rpsL	-165T>C	1	non-resistant associated
RL026b	1917972	Rv1694	tlyA	33A>G	1	non-resistant associated
RL026b	4242643	Rv3793	embC	2781C>T	1	non-resistant associated
RL026b	4326739	Rv3854c	ethA	735G>C	1	non-resistant associated
RL026b	4328004	Rv3855	ethR	456G>A	0.987	non-resistant associated
RL027a	6446	Rv0005	gyrB	Ala403Ser	1	non-resistant associated
RL027a	7362	Rv0006	gyrA	Glu21Gln	1	non-resistant associated
RL027a	7585	Rv0006	gyrA	Ser95Thr	1	non-resistant associated
RL027a	9143	Rv0006	gyrA	1842T>C	1	non-resistant associated
RL027a	9304	Rv0006	gyrA	Gly668Asp	1	non-resistant associated
RL027a	9566	Rv0006	gyrA	2265C>T	1	non-resistant associated
RL027a	763031	Rv0667	rpoB	3225T>C	1	non-resistant associated
RL027a	781395	Rv0682	rpsL	-165T>C	1	non-resistant associated
RL027a	1417554	Rv1267c	embR	-207C>G	1	non-resistant associated
RL027a	1673338	Rv1483	fabG1	-102G>A	1	non-resistant associated
RL027a	1917972	Rv1694	tlyA	33A>G	1	non-resistant associated

Participant_ID	Chromosome_Position	Gene_locus	Gene	Mutation	Estimated_fraction	Drug_resistant_to
RL027a	2154724	Rv1908c	katG	Arg463Leu	1	non-resistant associated
RL027a	2518132	Rv2245	kasA	18C>T	1	non-resistant associated
RL027a	4240671	Rv3793	embC	Thr270Ile	1	non-resistant associated
RL027a	4242643	Rv3793	embC	2781C>T	1	non-resistant associated
RL027a	4244220	Rv3794	embA	988C>T	1	non-resistant associated
RL027a	4244635	Rv3794	embA	Val468Ala	1	non-resistant associated
RL027a	4245147	Rv3794	embA	Pro639Ser	0.982	non-resistant associated
RL027a	4247646	Rv3795	embB	Glu378Ala	1	non-resistant associated
RL027a	4327103	Rv3854c	ethA	Gly124Asp	1	non-resistant associated
RL027a	4327355	Rv3854c	ethA	Ser40Phe	1	non-resistant associated
RL027a	4407588	Rv3919c	gid	615T>C	1	non-resistant associated
RL027b	6446	Rv0005	gyrB	Ala403Ser	1	non-resistant associated
RL027b	7362	Rv0006	gyrA	Glu21Gln	1	non-resistant associated
RL027b	7585	Rv0006	gyrA	Ser95Thr	0.989	non-resistant associated
RL027b	9143	Rv0006	gyrA	1842T>C	0.968	non-resistant associated
RL027b	9304	Rv0006	gyrA	Gly668Asp	1	non-resistant associated
RL027b	9566	Rv0006	gyrA	2265C>T	0.989	non-resistant associated
RL027b	763031	Rv0667	rpoB	3225T>C	1	non-resistant associated
RL027b	781395	Rv0682	rpsL	-165T>C	1	non-resistant associated
RL027b	1417554	Rv1267c	embR	-207C>G	0.987	non-resistant associated
RL027b	1673338	Rv1483	fabG1	-102G>A	1	non-resistant associated
RL027b	1917972	Rv1694	tlyA	33A>G	1	non-resistant associated
RL027b	2154724	Rv1908c	katG	Arg463Leu	1	non-resistant associated
RL027b	2518132	Rv2245	kasA	18C>T	0.976	non-resistant associated
RL027b	4240671	Rv3793	embC	Thr270Ile	0.972	non-resistant associated
RL027b	4242643	Rv3793	embC	2781C>T	1	non-resistant associated
RL027b	4244220	Rv3794	embA	988C>T	1	non-resistant associated
RL027b	4244635	Rv3794	embA	Val468Ala	1	non-resistant associated
RL027b	4245147	Rv3794	embA	Pro639Ser	1	non-resistant associated

Participant_ID	Chromosome_Position	Gene_locus	Gene	Mutation	Estimated_fraction	Drug_resistant_to
RL027b	4247646	Rv3795	embB	Glu378Ala	1	non-resistant associated
RL027b	4327103	Rv3854c	ethA	Gly124Asp	0.972	non-resistant associated
RL027b	4327355	Rv3854c	ethA	Ser40Phe	0.973	non-resistant associated
RL027b	4407588	Rv3919c	gid	615T>C	1	non-resistant associated
RL028a	2289091	Rv2043c	pncA	His51Tyr	0.986	pyrazinamide
RL028a	781687	Rv0682	rpsL	Lys43Arg	0.986	streptomycin
RL028a	6446	Rv0005	gyrB	Ala403Ser	1	non-resistant associated
RL028a	7362	Rv0006	gyrA	Glu21Gln	1	non-resistant associated
RL028a	7585	Rv0006	gyrA	Ser95Thr	1	non-resistant associated
RL028a	9143	Rv0006	gyrA	1842T>C	1	non-resistant associated
RL028a	9304	Rv0006	gyrA	Gly668Asp	1	non-resistant associated
RL028a	9566	Rv0006	gyrA	2265C>T	0.955	non-resistant associated
RL028a	763031	Rv0667	rpoB	3225T>C	0.988	non-resistant associated
RL028a	781395	Rv0682	rpsL	-165T>C	1	non-resistant associated
RL028a	781444	Rv0682	rpsL	-116C>T	0.968	non-resistant associated
RL028a	1417554	Rv1267c	embR	-207C>G	0.988	non-resistant associated
RL028a	1673338	Rv1483	fabG1	-102G>A	1	non-resistant associated
RL028a	1917972	Rv1694	tlyA	33A>G	1	non-resistant associated
RL028a	2154724	Rv1908c	katG	Arg463Leu	1	non-resistant associated
RL028a	2518132	Rv2245	kasA	18C>T	1	non-resistant associated
RL028a	4240671	Rv3793	embC	Thr270Ile	1	non-resistant associated
RL028a	4242643	Rv3793	embC	2781C>T	1	non-resistant associated
RL028a	4244220	Rv3794	embA	988C>T	1	non-resistant associated
RL028a	4244635	Rv3794	embA	Val468Ala	0.985	non-resistant associated
RL028a	4245147	Rv3794	embA	Pro639Ser	1	non-resistant associated
RL028a	4247646	Rv3795	embB	Glu378Ala	0.986	non-resistant associated
RL028a	4326928	Rv3854c	ethA	546G>A	1	non-resistant associated
RL028a	4327059	Rv3854c	ethA	Gly139Cys	0.977	non-resistant associated
RL028a	4327103	Rv3854c	ethA	Gly124Asp	0.989	non-resistant associated

Participant_ID	Chromosome_Position	Gene_locus	Gene	Mutation	Estimated_fraction	Drug_resistant_to
RL028a	4407588	Rv3919c	gid	615T>C	1	non-resistant associated
RL028b	2289091	Rv2043c	pncA	His51Tyr	1	pyrazinamide
RL028b	781687	Rv0682	rpsL	Lys43Arg	1	streptomycin
RL028b	6446	Rv0005	gyrB	Ala403Ser	1	non-resistant associated
RL028b	7362	Rv0006	gyrA	Glu21Gln	1	non-resistant associated
RL028b	7585	Rv0006	gyrA	Ser95Thr	1	non-resistant associated
RL028b	9143	Rv0006	gyrA	1842T>C	1	non-resistant associated
RL028b	9304	Rv0006	gyrA	Gly668Asp	1	non-resistant associated
RL028b	9566	Rv0006	gyrA	2265C>T	1	non-resistant associated
RL028b	763031	Rv0667	rpoB	3225T>C	1	non-resistant associated
RL028b	781395	Rv0682	rpsL	-165T>C	1	non-resistant associated
RL028b	781444	Rv0682	rpsL	-116C>T	1	non-resistant associated
RL028b	1417554	Rv1267c	embR	-207C>G	1	non-resistant associated
RL028b	1673338	Rv1483	fabG1	-102G>A	1	non-resistant associated
RL028b	1917972	Rv1694	tlyA	33A>G	1	non-resistant associated
RL028b	2154724	Rv1908c	katG	Arg463Leu	1	non-resistant associated
RL028b	2518132	Rv2245	kasA	18C>T	1	non-resistant associated
RL028b	4240671	Rv3793	embC	Thr270Ile	1	non-resistant associated
RL028b	4242643	Rv3793	embC	2781C>T	1	non-resistant associated
RL028b	4244220	Rv3794	embA	988C>T	1	non-resistant associated
RL028b	4244635	Rv3794	embA	Val468Ala	1	non-resistant associated
RL028b	4245147	Rv3794	embA	Pro639Ser	1	non-resistant associated
RL028b	4247646	Rv3795	embB	Glu378Ala	1	non-resistant associated
RL028b	4326928	Rv3854c	ethA	546G>A	0.983	non-resistant associated
RL028b	4327059	Rv3854c	ethA	Gly139Cys	1	non-resistant associated
RL028b	4327103	Rv3854c	ethA	Gly124Asp	1	non-resistant associated
RL028b	4407588	Rv3919c	gid	615T>C	1	non-resistant associated
RL029a	7362	Rv0006	gyrA	Glu21Gln	1	non-resistant associated
RL029a	7585	Rv0006	gyrA	Ser95Thr	1	non-resistant associated

Participant_ID	Chromosome_Position	Gene_locus	Gene	Mutation	Estimated_fraction	Drug_resistant_to
RL029a	9304	Rv0006	gyrA	Gly668Asp	1	non-resistant associated
RL029a	9557	Rv0006	gyrA	2256G>C	1	non-resistant associated
RL029a	765150	Rv0668	rpoC	Gly594Glu	1	non-resistant associated
RL029a	781395	Rv0682	rpsL	-165T>C	1	non-resistant associated
RL029a	781421	Rv0682	rpsL	-139C>A	0.989	non-resistant associated
RL029a	1917972	Rv1694	tlyA	33A>G	1	non-resistant associated
RL029a	4242643	Rv3793	embC	2781C>T	1	non-resistant associated
RL029a	4242803	Rv3793	embC	Val981Leu	1	non-resistant associated
RL029a	4246541	Rv3795	embB	Ser10Arg	1	non-resistant associated
RL029b	7362	Rv0006	gyrA	Glu21Gln	1	non-resistant associated
RL029b	7585	Rv0006	gyrA	Ser95Thr	1	non-resistant associated
RL029b	9304	Rv0006	gyrA	Gly668Asp	1	non-resistant associated
RL029b	9557	Rv0006	gyrA	2256G>C	1	non-resistant associated
RL029b	765150	Rv0668	rpoC	Gly594Glu	0.987	non-resistant associated
RL029b	781395	Rv0682	rpsL	-165T>C	1	non-resistant associated
RL029b	781421	Rv0682	rpsL	-139C>A	0.987	non-resistant associated
RL029b	1917972	Rv1694	tlyA	33A>G	1	non-resistant associated
RL029b	4242643	Rv3793	embC	2781C>T	1	non-resistant associated
RL029b	4242803	Rv3793	embC	Val981Leu	0.978	non-resistant associated
RL029b	4246541	Rv3795	embB	Ser10Arg	0.954	non-resistant associated
RL030a	4408100	Rv3919c	gid	102 102del	1	streptomycin
RL030a	7362	Rv0006	gyrA	Glu21Gln	1	non-resistant associated
RL030a	7585	Rv0006	gyrA	Ser95Thr	1	non-resistant associated
RL030a	7762	Rv0006	gyrA	Pro154Arg	0.976	non-resistant associated
RL030a	9304	Rv0006	gyrA	Gly668Asp	1	non-resistant associated
RL030a	781395	Rv0682	rpsL	-165T>C	1	non-resistant associated
RL030a	1917972	Rv1694	tlyA	33A>G	1	non-resistant associated
RL030a	4242643	Rv3793	embC	2781C>T	1	non-resistant associated
RL030a	4326739	Rv3854c	ethA	735G>C	0.98	non-resistant associated

Participant_ID	Chromosome_Position	Gene_locus	Gene	Mutation	Estimated_fraction	Drug_resistant_to
RL030a	4328004	Rv3855	ethR	456G>A	0.982	non-resistant associated
RL030b	4408100	Rv3919c	gid	102 102del	0.953	streptomycin
RL030b	7362	Rv0006	gyrA	Glu21Gln	1	non-resistant associated
RL030b	7585	Rv0006	gyrA	Ser95Thr	1	non-resistant associated
RL030b	7762	Rv0006	gyrA	Pro154Arg	1	non-resistant associated
RL030b	9304	Rv0006	gyrA	Gly668Asp	1	non-resistant associated
RL030b	781395	Rv0682	rpsL	-165T>C	1	non-resistant associated
RL030b	1917972	Rv1694	tlyA	33A>G	1	non-resistant associated
RL030b	4242643	Rv3793	embC	2781C>T	1	non-resistant associated
RL030b	4326739	Rv3854c	ethA	735G>C	0.985	non-resistant associated
RL030b	4328004	Rv3855	ethR	456G>A	0.979	non-resistant associated
RL031a	6446	Rv0005	gyrB	Ala403Ser	1	non-resistant associated
RL031a	7362	Rv0006	gyrA	Glu21Gln	1	non-resistant associated
RL031a	7585	Rv0006	gyrA	Ser95Thr	1	non-resistant associated
RL031a	9143	Rv0006	gyrA	1842T>C	1	non-resistant associated
RL031a	9304	Rv0006	gyrA	Gly668Asp	1	non-resistant associated
RL031a	9566	Rv0006	gyrA	2265C>T	1	non-resistant associated
RL031a	763031	Rv0667	rpoB	3225T>C	1	non-resistant associated
RL031a	781395	Rv0682	rpsL	-165T>C	1	non-resistant associated
RL031a	1417554	Rv1267c	embR	-207C>G	1	non-resistant associated
RL031a	1673338	Rv1483	fabG1	-102G>A	1	non-resistant associated
RL031a	1917972	Rv1694	tlyA	33A>G	1	non-resistant associated
RL031a	2154724	Rv1908c	katG	Arg463Leu	1	non-resistant associated
RL031a	2518132	Rv2245	kasA	18C>T	1	non-resistant associated
RL031a	2726261	Rv2428	ahpC	69G>T	1	non-resistant associated
RL031a	4240671	Rv3793	embC	Thr270Ile	1	non-resistant associated
RL031a	4242643	Rv3793	embC	2781C>T	1	non-resistant associated
RL031a	4244220	Rv3794	embA	988C>T	1	non-resistant associated
RL031a	4244635	Rv3794	embA	Val468Ala	1	non-resistant associated

Participant_ID	Chromosome_Position	Gene_locus	Gene	Mutation	Estimated_fraction	Drug_resistant_to
RL031a	4245147	Rv3794	embA	Pro639Ser	1	non-resistant associated
RL031a	4247646	Rv3795	embB	Glu378Ala	1	non-resistant associated
RL031a	4326928	Rv3854c	ethA	546G>A	1	non-resistant associated
RL031a	4327103	Rv3854c	ethA	Gly124Asp	1	non-resistant associated
RL031a	4407588	Rv3919c	gid	615T>C	1	non-resistant associated
RL031b	6446	Rv0005	gyrB	Ala403Ser	0.984	non-resistant associated
RL031b	7362	Rv0006	gyrA	Glu21Gln	1	non-resistant associated
RL031b	7585	Rv0006	gyrA	Ser95Thr	1	non-resistant associated
RL031b	9143	Rv0006	gyrA	1842T>C	0.978	non-resistant associated
RL031b	9304	Rv0006	gyrA	Gly668Asp	1	non-resistant associated
RL031b	9566	Rv0006	gyrA	2265C>T	1	non-resistant associated
RL031b	763031	Rv0667	rpoB	3225T>C	1	non-resistant associated
RL031b	781395	Rv0682	rpsL	-165T>C	1	non-resistant associated
RL031b	1417554	Rv1267c	embR	-207C>G	1	non-resistant associated
RL031b	1673338	Rv1483	fabG1	-102G>A	1	non-resistant associated
RL031b	1917972	Rv1694	tlyA	33A>G	1	non-resistant associated
RL031b	2154724	Rv1908c	katG	Arg463Leu	0.983	non-resistant associated
RL031b	2518132	Rv2245	kasA	18C>T	1	non-resistant associated
RL031b	2726261	Rv2428	ahpC	69G>T	1	non-resistant associated
RL031b	4240671	Rv3793	embC	Thr270Ile	1	non-resistant associated
RL031b	4242643	Rv3793	embC	2781C>T	1	non-resistant associated
RL031b	4244220	Rv3794	embA	988C>T	1	non-resistant associated
RL031b	4244635	Rv3794	embA	Val468Ala	1	non-resistant associated
RL031b	4245147	Rv3794	embA	Pro639Ser	1	non-resistant associated
RL031b	4247646	Rv3795	embB	Glu378Ala	1	non-resistant associated
RL031b	4326928	Rv3854c	ethA	546G>A	0.986	non-resistant associated
RL031b	4327103	Rv3854c	ethA	Gly124Asp	1	non-resistant associated
RL031b	4407588	Rv3919c	gid	615T>C	1	non-resistant associated
RL032a	7362	Rv0006	gyrA	Glu21Gln	1	non-resistant associated

Participant_ID	Chromosome_Position	Gene_locus	Gene	Mutation	Estimated_fraction	Drug_resistant_to
RL032a	7585	Rv0006	gyrA	Ser95Thr	1	non-resistant associated
RL032a	9304	Rv0006	gyrA	Gly668Asp	1	non-resistant associated
RL032a	781395	Rv0682	rpsL	-165T>C	1	non-resistant associated
RL032a	1917972	Rv1694	tlyA	33A>G	1	non-resistant associated
RL032a	2289037	Rv2043c	pncA	Pro69Ser	1	non-resistant associated
RL032a	4242550	Rv3793	embC	2688C>G	1	non-resistant associated
RL032a	4242643	Rv3793	embC	2781C>T	1	non-resistant associated
RL032a	4326739	Rv3854c	ethA	735G>C	1	non-resistant associated
RL032a	4328004	Rv3855	ethR	456G>A	1	non-resistant associated
RL032b	7362	Rv0006	gyrA	Glu21Gln	1	non-resistant associated
RL032b	7585	Rv0006	gyrA	Ser95Thr	1	non-resistant associated
RL032b	9304	Rv0006	gyrA	Gly668Asp	1	non-resistant associated
RL032b	781395	Rv0682	rpsL	-165T>C	1	non-resistant associated
RL032b	1917972	Rv1694	tlyA	33A>G	1	non-resistant associated
RL032b	2289037	Rv2043c	pncA	Pro69Ser	0.98	non-resistant associated
RL032b	4242550	Rv3793	embC	2688C>G	1	non-resistant associated
RL032b	4242643	Rv3793	embC	2781C>T	1	non-resistant associated
RL032b	4326739	Rv3854c	ethA	735G>C	1	non-resistant associated
RL032b	4328004	Rv3855	ethR	456G>A	1	non-resistant associated
RL033a	7362	Rv0006	gyrA	Glu21Gln	1	non-resistant associated
RL033a	7585	Rv0006	gyrA	Ser95Thr	1	non-resistant associated
RL033a	7762	Rv0006	gyrA	Pro154Arg	1	non-resistant associated
RL033a	9304	Rv0006	gyrA	Gly668Asp	1	non-resistant associated
RL033a	781395	Rv0682	rpsL	-165T>C	1	non-resistant associated
RL033a	1917972	Rv1694	tlyA	33A>G	1	non-resistant associated
RL033a	4242643	Rv3793	embC	2781C>T	1	non-resistant associated
RL033a	4326739	Rv3854c	ethA	735G>C	1	non-resistant associated
RL033a	4328004	Rv3855	ethR	456G>A	0.988	non-resistant associated
RL033b	7362	Rv0006	gyrA	Glu21Gln	1	non-resistant associated

Participant_ID	Chromosome_Position	Gene_locus	Gene	Mutation	Estimated_fraction	Drug_resistant_to
RL033b	7585	Rv0006	gyrA	Ser95Thr	1	non-resistant associated
RL033b	7762	Rv0006	gyrA	Pro154Arg	0.977	non-resistant associated
RL033b	9304	Rv0006	gyrA	Gly668Asp	1	non-resistant associated
RL033b	781395	Rv0682	rpsL	-165T>C	1	non-resistant associated
RL033b	1917972	Rv1694	tlyA	33A>G	1	non-resistant associated
RL033b	2726736	Rv2428	ahpC	Asp182Asn	0.956	non-resistant associated
RL033b	4242643	Rv3793	embC	2781C>T	1	non-resistant associated
RL033b	4326739	Rv3854c	ethA	735G>C	0.988	non-resistant associated
RL033b	4328004	Rv3855	ethR	456G>A	0.99	non-resistant associated
RL034a	7362	Rv0006	gyrA	Glu21Gln	1	non-resistant associated
RL034a	781395	Rv0682	rpsL	-165T>C	1	non-resistant associated
RL034a	1474001	rrl	rrl	r.344c>t	1	non-resistant associated
RL034a	1917972	Rv1694	tlyA	33A>G	1	non-resistant associated
RL034a	4242643	Rv3793	embC	2781C>T	1	non-resistant associated
RL034b	6446	Rv0005	gyrB	Ala403Ser	0.891	non-resistant associated
RL034b	7362	Rv0006	gyrA	Glu21Gln	1	non-resistant associated
RL034b	7585	Rv0006	gyrA	Ser95Thr	1	non-resistant associated
RL034b	9143	Rv0006	gyrA	1842T>C	0.978	non-resistant associated
RL034b	9304	Rv0006	gyrA	Gly668Asp	1	non-resistant associated
RL034b	9566	Rv0006	gyrA	2265C>T	0.983	non-resistant associated
RL034b	763031	Rv0667	rpoB	3225T>C	1	non-resistant associated
RL034b	763280	Rv0667	rpoB	3474C>T	0.971	non-resistant associated
RL034b	781395	Rv0682	rpsL	-165T>C	1	non-resistant associated
RL034b	1417554	Rv1267c	embR	-207C>G	0.935	non-resistant associated
RL034b	1673338	Rv1483	fabG1	-102G>A	0.967	non-resistant associated
RL034b	1917972	Rv1694	tlyA	33A>G	1	non-resistant associated
RL034b	2154724	Rv1908c	katG	Arg463Leu	0.98	non-resistant associated
RL034b	2518132	Rv2245	kasA	18C>T	0.984	non-resistant associated
RL034b	4240671	Rv3793	embC	Thr270Ile	0.977	non-resistant associated

Participant_ID	Chromosome_Position	Gene_locus	Gene	Mutation	Estimated_fraction	Drug_resistant_to
RL034b	4241138	Rv3793	embC	Ala426Pro	1	non-resistant associated
RL034b	4242643	Rv3793	embC	2781C>T	1	non-resistant associated
RL034b	4244220	Rv3794	embA	988C>T	0.98	non-resistant associated
RL034b	4244635	Rv3794	embA	Val468Ala	0.945	non-resistant associated
RL034b	4245147	Rv3794	embA	Pro639Ser	0.981	non-resistant associated
RL034b	4247646	Rv3795	embB	Glu378Ala	0.954	non-resistant associated
RL034b	4326928	Rv3854c	ethA	546G>A	0.985	non-resistant associated
RL034b	4327103	Rv3854c	ethA	Gly124Asp	0.984	non-resistant associated
RL034b	4407588	Rv3919c	gid	615T>C	0.949	non-resistant associated
RL035a	7362	Rv0006	gyrA	Glu21Gln	1	non-resistant associated
RL035a	7585	Rv0006	gyrA	Ser95Thr	1	non-resistant associated
RL035a	9304	Rv0006	gyrA	Gly668Asp	1	non-resistant associated
RL035a	763031	Rv0667	rpoB	3225T>C	0.988	non-resistant associated
RL035a	781395	Rv0682	rpsL	-165T>C	1	non-resistant associated
RL035a	1834177	Rv1630	rpsA	636A>C	1	non-resistant associated
RL035a	1917972	Rv1694	tlyA	33A>G	0.987	non-resistant associated
RL035a	2154724	Rv1908c	katG	Arg463Leu	1	non-resistant associated
RL035a	4242643	Rv3793	embC	2781C>T	1	non-resistant associated
RL035a	4243460	Rv3794	embA	228C>T	1	non-resistant associated
RL035a	4326676	Rv3854c	ethA	Ser266Arg	0.988	non-resistant associated
RL035a	4326977	Rv3854c	ethA	His166Arg	1	non-resistant associated
RL035a	4407588	Rv3919c	gid	615T>C	0.985	non-resistant associated
RL035a	4407927	Rv3919c	gid	Glu92Asp	0.966	non-resistant associated
RL035b	7362	Rv0006	gyrA	Glu21Gln	1	non-resistant associated
RL035b	7585	Rv0006	gyrA	Ser95Thr	1	non-resistant associated
RL035b	9304	Rv0006	gyrA	Gly668Asp	1	non-resistant associated
RL035b	763031	Rv0667	rpoB	3225T>C	1	non-resistant associated
RL035b	781395	Rv0682	rpsL	-165T>C	1	non-resistant associated
RL035b	1834177	Rv1630	rpsA	636A>C	1	non-resistant associated

Participant_ID	Chromosome_Position	Gene_locus	Gene	Mutation	Estimated_fraction	Drug_resistant_to
RL035b	1917972	Rv1694	tlyA	33A>G	1	non-resistant associated
RL035b	2154724	Rv1908c	katG	Arg463Leu	1	non-resistant associated
RL035b	4242643	Rv3793	embC	2781C>T	1	non-resistant associated
RL035b	4243460	Rv3794	embA	228C>T	0.987	non-resistant associated
RL035b	4326676	Rv3854c	ethA	Ser266Arg	1	non-resistant associated
RL035b	4326977	Rv3854c	ethA	His166Arg	1	non-resistant associated
RL035b	4407588	Rv3919c	gid	615T>C	1	non-resistant associated
RL035b	4407927	Rv3919c	gid	Glu92Asp	1	non-resistant associated
RL019a	7362	Rv0006	gyrA	p.Glu21Gln	1	non-resistant associated
RL019a	7585	Rv0006	gyrA	p.Ser95Thr	1	non-resistant associated
RL019a	9304	Rv0006	gyrA	p.Gly668Asp	1	non-resistant associated
RL019a	9557	Rv0006	gyrA	c.2256G>C	1	non-resistant associated
RL019a	765150	Rv0668	rpoC	p.Gly594Glu	0.986	non-resistant associated
RL019a	781395	Rv0682	rpsL	c.-165T>C	1	non-resistant associated
RL019a	781421	Rv0682	rpsL	c.-139C>A	1	non-resistant associated
RL019a	1917972	Rv1694	tlyA	c.33A>G	1	non-resistant associated
RL019a	3086788	Rv2780	ald	c.-32T>C	1	non-resistant associated
RL019a	4242643	Rv3793	embC	c.2781C>T	1	non-resistant associated
RL019a	4242803	Rv3793	embC	p.Val981Leu	0.98	non-resistant associated
RL016b	761161	Rv0667	rpoB	p.Leu452Pro	1	rifampicin
RL016b	1673432	Rv1483	fabG1	c.-8T>C	0.984	ethionamide,isoniazid
RL016b	2155168	Rv1908c	katG	p.Ser315Thr	0.986	isoniazid
RL016b	2289103	Rv2043c	pncA	p.Thr47Ala	0.571	pyrazinamide
RL016b	4247513	Rv3795	embB	p.Tyr334His	0.988	ethambutol
RL016b	4248003	Rv3795	embB	p.Gln497Arg	0.987	ethambutol
RL016b	6446	Rv0005	gyrB	p.Ala403Ser	1	non-resistant associated
RL016b	7362	Rv0006	gyrA	p.Glu21Gln	1	non-resistant associated
RL016b	7585	Rv0006	gyrA	p.Ser95Thr	1	non-resistant associated
RL016b	9143	Rv0006	gyrA	c.1842T>C	0.987	non-resistant associated

Participant_ID	Chromosome_Position	Gene_locus	Gene	Mutation	Estimated_fraction	Drug_resistant_to
RL016b	9304	Rv0006	gyrA	p.Gly668Asp	1	non-resistant associated
RL016b	760969	Rv0667	rpoB	p.Ser388Leu	1	non-resistant associated
RL016b	761723	Rv0667	rpoB	p.Glu639Asp	1	non-resistant associated
RL016b	763031	Rv0667	rpoB	c.3225T>C	1	non-resistant associated
RL016b	779209	Rv0678	Rv0678	p.Leu74Met	1	non-resistant associated
RL016b	781395	Rv0682	rpsL	c.-165T>C	1	non-resistant associated
RL016b	1417554	Rv1267c	embR	c.-207C>G	1	non-resistant associated
RL016b	1674434	Rv1484	inhA	p.Val78Ala	1	non-resistant associated
RL016b	1917972	Rv1694	tlyA	c.33A>G	1	non-resistant associated
RL016b	2154724	Rv1908c	katG	p.Arg463Leu	1	non-resistant associated
RL016b	2155503	Rv1908c	katG	c.609G>A	0.989	non-resistant associated
RL016b	2289232	Rv2043c	pncA	p.Leu4Val	1	non-resistant associated
RL016b	2518132	Rv2245	kasA	c.18C>T	1	non-resistant associated
RL016b	3086788	Rv2780	ald	c.-32T>C	1	non-resistant associated
RL016b	3087084	Rv2780	ald	c.266 266del	1	non-resistant associated
RL016b	3841662	Rv3423c	alr	c.-242A>G	1	non-resistant associated
RL016b	3841663	Rv3423c	alr	c.-243G>A	1	non-resistant associated
RL016b	4240671	Rv3793	embC	p.Thr270Ile	0.986	non-resistant associated
RL016b	4241843	Rv3793	embC	p.Leu661Ile	1	non-resistant associated
RL016b	4242643	Rv3793	embC	c.2781C>T	1	non-resistant associated
RL016b	4244220	Rv3794	embA	c.988C>T	1	non-resistant associated
RL016b	4244379	Rv3794	embA	p.Pro383Ser	0.987	non-resistant associated
RL016b	4246864	Rv3795	embB	c.351C>T	1	non-resistant associated
RL016b	4247646	Rv3795	embB	p.Glu378Ala	0.99	non-resistant associated
RL016b	4326465	Rv3854c	ethA	p.Ile337Val	1	non-resistant associated
RL016b	4326711	Rv3854c	ethA	p.Lys255Gln	1	non-resistant associated
RL016b	4327080	Rv3854c	ethA	132E>132*	1	non-resistant associated
RL016b	4407588	Rv3919c	gid	c.615T>C	1	non-resistant associated

Participant_ID	Chromosome_Position	Gene_locus	Gene	Mutation	Estimated_fraction	Drug_resistant_to
RL016b	4407980	Rv3919c	gid	p.Pro75Ala	0.978	non-resistant associated
FT002b	7362	Rv0006	gyrA	p.Glu21Gln	1	non-resistant associated
FT002b	7585	Rv0006	gyrA	p.Ser95Thr	1	non-resistant associated
FT002b	9304	Rv0006	gyrA	p.Gly668Asp	0.984	non-resistant associated
FT002b	781395	Rv0682	rpsL	c.-165T>C	1	non-resistant associated
FT002b	1917972	Rv1694	tlyA	c.33A>G	1	non-resistant associated
FT002b	4242643	Rv3793	embC	c.2781C>T	1	non-resistant associated
FT002b	4326739	Rv3854c	ethA	c.735G>C	1	non-resistant associated
FT002b	4328004	Rv3855	ethR	c.456G>A	1	non-resistant associated

Single nucleotide polymorphisms (SNPs) in coding regions are annotated using the reference amino acid, codon number and alternative amino acid. SNPs in non-coding regions (i.e. RNA genes and intergenic regions) are annotated using the reference nucleotide, gene coordinate and alternative nucleotide (e.g. A1401G in rrs or C-37A in eis promoter). Indels are annotated using the reference VCF allele, gene coordinate and alternative VCF allele (e.g. T902TA insertion in katG)

Supplementary table 3. List of MIRU-VNTR allelic information for all participants

Participant ID	MIRU 10	MIRU 16	MIRU 26	MIRU 40	QUB2163b	ETRA (2165)	QUB26 (4052)	QUB4156	VNTR 424	VNTR 577	VNTR 580	VNTR 1955	VNTR 2401	VNTR 3192	VNTR 3690
FT001a	3	3	1	3	3	3	4	2	2	4	2	3	2	3	4
FT001b	3	3	1	3	3	3	4	2	2	4	2	3	2	3	4
FT002a	3	3	5	4	4	4	5	2	2	4	2	3	2	3	4
FT002b	3	3	6	3	4	4	5	2	2	4	2	3	2	3	4
FT003a	3	3	5	3	6	4	5	2	2	4	2	3	2	2	
FT003b	3	3	5	3	6	4	5	2	2	4	2	3	2	2	
FT004a	3	3	5	3	5	4	5	2	2	4	2	3	2	3	
FT004b	3	3	5	3	5	4	5	2	2	4	2	3	2	3	
FT004c	3	3	5	3	5	4	5	2	2	4	2	3	2	3	
FT006a	6	4	7	3	2	4	7	4							
FT006b	2	3	5	1	6	4	5	2	2	4	2	3	2	3	3
FT007a	3	3	6	3	4	4	7	2	4	4	2	4	4	5	3
FT007b	3	3	6	3		3	7	2	4	4	2	4	4	5	3
FT012a	6	3	5	4	5	3	5	3	2	3	2	3	4	3	3
FT012b	6	3	5	4	5	3	5	3	2	3	2	3	4	3	3
FT012c	3	2	5	4	3	3	5	3	2	3	2	3	4	3	3
FT012d	7	5	4	1		4	2	3	2	3	2	2	4	2	3
FT013a	3	3	5	3	6	4	5	2	2	4	2	3	2	2	18
FT013b	3	3	5	3	6	4	5	2	2	4	2	3	2	2	18
FT014a	3	3	6	3	6	4	7	2	4	4	2	4	4	5	3
FT014b	3	3	6	3	6	4	7	2	4	4	2	4	4	5	3
FT015a	3	3	5	3	6	4	5	2	2	4	2	3	2	2	18
FT015b	3	3	5	3	6	4	5	2	2	4	2	3	2	2	18
FT016a	3	3	4	1	2	3	4	2	2	4	2	3	2	3	4
FT016b	3	3	4	1	2	3	4	2	2	4	2	3	2	3	4
FT016c	3	3	4	1	2	3	4	2	2	4	2	3	2	3	4
RL001a	3	3	5	3	6	4	5	2	2	4	2	3	2	3	11
RL001b	3	3	5	3	6	4	5	2	2	4	2	3	2	3	11
RL002a	2	3	4	3	2	3	6	2	2	5	2	3	2	3	7
RL002b	2	3	4	3	2	3	5	2							
RL003a	7	2	4	1	4	7	6	1	2	5	2	3	4	5	5
RL003b	3	2	5	3	4	3	5	2	2	4	2	3	2	3	8
RL004a	3	3	5	3	6	4	5	2	2	4	2	3	2	2	16
RL004b	3	3	5	3	6	4	5	2	2	4	2	3	2	2	16
RL005b	5	5	4	1	1	4	3	3	3	4	2	2	4	2	3

Participant ID	MIRU 10	MIRU 16	MIRU 26	MIRU 40	QUB2163b	ETRA (2165)	QUB26 (4052)	QUB4156	VNTR 424	VNTR 577	VNTR 580	VNTR 1955	VNTR 2401	VNTR 3192	VNTR 3690
RL006a	3	4	4	3	3	4	4	2	3	4	2	3	2	3	6
RL007a	4	4	4	1	5	4	2	3	1	4	2	2	4	2	3
RL007b	4	4	4	1		4	2	3	1	4	2	2	4	2	2
RL008a	3	1	5	4	2	2	5	2	2	5	2	2	2	3	3
RL008b	3	3	5	3	4	2	4	2	2	4	2	3	2	3	4
RL009a	3	3	5	3	5	4	4	2	2	4	2	3	2	3	
RL009b	3	3	5	3	6	4	4	2	2	4	2	3	2	3	3
RL010a	3	3	5	3	6	4	5	2	2	4	2	3	2	2	17
RL010b	3	3	5	3	6	4	5	2	2	4	2	3	2	2	
RL011a	3	1	5	3	5	3	5	2	3	4	2	2	4	2	3
RL011b	2	2	5	3	3	3		2	2	4	2	3	2	3	
RL011c	2	2	5	3	3	3	5	2	2	4	2	3	2	3	10
RL012a	3	3	6	3	6	4	7	2	4	4	2	4	4	5	3
RL012b	3	3	6	3	6	4	7	2	4	4	2	4	4	5	3
RL013a	3	3	5	3	6	4	5	2	2	4	2	3	2	2	16
RL013b	3	3	5	3	6	4	5	2	2	4	2	3	2	2	16
RL013c															
RL014a	3	3	5	3	3	2	4	2	2	4	2	3	2	3	9
RL014b	3	3	5	3	3	2	4	2	2	4	2	3	2	3	9
RL015a	3	3	5	3	6	4	5	2	2	4	2	3	2	3	
RL015b	4	3	4	3	6	3	7	3	2	3	2	4	4	3	3
RL016a	4	3	4	2	5	6	7	3	3	5	2	4	4	5	4
RL016b	4	3	4	2	5	6	7	3	3	5	2	4	4	5	4
RL017a															
RL017b		1	1	3	3							0			0
RL018a	3	3	5	3	6	4	5	2	2	4	2	3	2	2(+3)	3
RL018b	3	3	4		3	3		1	2	3	2	3	2	3	3
RL019a	3	4	4	2	3	3	6	1							
RL019b															
RL020a	6	3	5	3	5	3	5	3	2	3	2	3	4	3	3
RL020b	6	3	5	3	5	3	5	2	2	3	2	3	4	3	3
RL021a	3	3	5	3	6	4	5	2	2	4	2	3	2	2	3
RL021b	3	3	5	3	6	4	5	2	2	4	2	3	2	2	2
RL022a	3	3	5	3	6	4	5	2	2	4	2	3	2	2	
RL022b	3	3	5	3	6	4	5	2	2	4	2	3	2	2	
RL022c	3	3	5	3	6	4	5	2	2	4	2	3	2	2	
RL023a	3	3	4	2	6	3	5	2	2	4	2	2	2	3	1
RL023b	3	3	4	2	6	3	5	2	2	4	2	2	2	3	1

Participant ID	MIRU 10	MIRU 16	MIRU 26	MIRU 40	QUB2163b	ETRA (2165)	QUB26 (4052)	QUB4156	VNTR 424	VNTR 577	VNTR 580	VNTR 1955	VNTR 2401	VNTR 3192	VNTR 3690
RL024a	2	3	5	1	6	4	5	2	2	4	2	3	2	3	2
RL024b	3	3	5	1	6	4	5	2	2	4	2	3	2	3	
RL025a	3	3	4	5	3	3	6	1	2	3	2	4	4	3	3
RL025b	3	3	4	5	3	3	6	1	2	3	2	4	4	3	3
RL026a	3	3	5	3	4	4	5	2	2	4	2	2	2	2	3
RL026b	3	3	4	3	4	4	5	2	2	4	2	3	2	3	4
RL027a	4	4	2	1		2	2	3	3	4	3	2	2	2	5
RL027b	4	4	2	1	-	2	2	3							
RL028a	4	4	4	1	6	4	3	3	3	4	2	2	4	2	3
RL028b	4	4	4	1		4	3	3	3	4	2	2	4	2	3
RL029a	3	3	4	6	3	3	6	1	2	3	2	4	4	3	3
RL029b	3	3	4	6	3	3	6	1	2	3	2	4	4	3	3
RL030a	3	3	5	3	6	4	5	2	2	4	2	3	2	3	2
RL030b	3	3	5	3	6	4	5	2	2	4	2	3	2	3	3
RL031a	4	4	4	1	2	4	2	3	1	4	2	2	2	2	3
RL031b	4	5	4	1	2	4	2	3	1	4	2	2	2	2	3
RL032a	3	3	5	3	6	4	5	2	2	4	2	3	2	3	1
RL032b	3	3	5	3	6	4	5	2	2	4	2	3	2	2	
RL033a	3	3	5	3	6	4	5	2	2	4	2	3	2	3	16
RL033b	3	3	5	3	6	4	5	2	2	4	2	3	2	3	
RL034a	4	4	2	1	6	4	2	2	1	4	2	2	4	2	3
RL034b	3	-	-	1	6	4	2	-							
RL035a	3	3	6	3	6	4	7	2	4	4	2	4	4	5	3
RL035b	3	3	6	3	6	4	5	2	4	4	2	4	4	5	3
RL036a	6	3	5	4	5	3	5	3	2	3	2	3	4	3	3
RL036b	6	3	5	4	5	3	5	3	2	3	2	3	4	3	3
